# The Evolving Puzzle of Autosomal *Versus* Y-linked Male Determination in *Musca domestica*

**DOI:** 10.1534/g3.114.014795

**Published:** 2014-12-31

**Authors:** Ronda L. Hamm, Richard P. Meisel, Jeffrey G. Scott

**Affiliations:** * Dow AgroSciences, Indianapolis, Indiana 46268; † Department of Biology and Biochemistry, University of Houston, Houston, Texas 77204; ‡ Department of Entomology, Comstock Hall, Cornell University, Ithaca, New York 14853

**Keywords:** sex determination, house fly, autosomal male, *Md-tra^D^*, genetics of sex

## Abstract

Sex determination is one of the most rapidly evolving developmental pathways, but the factors responsible for this fast evolution are not well resolved. The house fly, *Musca domestica*, is an ideal model for studying sex determination because house fly sex determination is polygenic and varies considerably between populations. Male house flies possess a male-determining locus, the M factor, which can be located on the Y or X chromosome or any of the five autosomes. There can be a single M or multiple M factors present in an individual male, in heterozygous or homozygous condition. Males with multiple copies of M skew the sex ratio toward the production of males. Potentially in response to these male-biased sex ratios, an allele of the gene *transformer*, *Md-tra^D^*, promotes female development in the presence of one or multiple M factors. There have been many studies to determine the linkage and frequency of these male determining factors and the frequency of *Md-tra^D^* chromosomes in populations from around the world. This review provides a summary of the information available to date regarding the patterns of distribution of autosomal, X-linked and Y-linked M factors, the relative frequencies of the linkage of M, the changes in frequencies found in field populations, and the fitness of males with autosomal M factors *vs.* Y-linked M. We evaluate this natural variation in the house fly sex determination pathway in light of models of the evolution of sex determination.

Sex determination is the initiation of a gene regulatory cascade responsible for the differential expression of genes between males and females, giving rise to reproductive traits and sexually dimorphic phenotypes. Paradoxically, even though sex determination is an essential developmental pathway required for fertility, sex determination pathways evolve extremely fast (Bull 1983; Marin and Baker 1998; Haag and Doty 2005). The genes or environmental cues responsible for the initiation of sex determination (master regulators) often differ between closely related species (Wilkins 1995; Graham *et al.* 2003). This evolutionary turnover in the initiation of sex determination pathways is contrasted by the use of conserved downstream components across distantly related taxa. For example, genes from the *doublesex/mab-3 related* (*Dmrt*) family are involved in sex determination pathways in vertebrates, insects, and round worms (Raymond *et al.* 1998; Haag and Doty 2005).

Multiple hypotheses have been put forth to explain the evolutionary turnover at the top of sex determination pathways. In one set of models, it was demonstrated that a novel sex determiner can invade a population if it is genetically linked to a beneficial allele (Charlesworth and Charlesworth 1980; Rice 1986). If the allele linked to the sex determiner confers a fitness benefit to one sex and is detrimental to the other sex (*i.e.*, it has a sexually antagonistic fitness effect), the new sex determiner is particularly likely to invade because it resolves the intersexual conflict by limiting the inheritance of the sexually antagonistic allele to the sex in which it is beneficial (Van Doorn and Kirkpatrick 2007, 2010). In another set of models, it was shown that a new sex-determining locus can invade a population if the sex ratio (relative number of breeding males and females) deviates from the equilibrium (often 1:1) (Eshel 1975; Bull and Charnov 1977; Bulmer and Bull 1982). In this case, the new sex determining locus produces a balanced sex ratio.

The house fly, *Musca domestica*, is a powerful model system for studying the genetics, molecular biology, and evolution of sex determination. The house fly has one of the most polymorphic sex determination pathways of any animal (Bull 1983; Dübendorfer 2001), and the past two decades have seen substantial advances in our understanding of the molecular regulation of the house fly sex determination pathway (Meise *et al.* 1998; Dübendorfer 2001; Hediger *et al.* 2004; Burghardt *et al.* 2005; Hediger *et al.* 2010; Meier *et al.* 2013). We highlight features of this polymorphism, and we describe how experiments on house flies have contributed toward our understanding of the evolution of sex determination. The house fly genome was sequenced recently (Scott *et al.* 2014a), which opens the door for improved understanding of sex determination in this species. We close with predictions for future insights that can be gleaned from technological advances in genomics and potential applications for control of house flies, which are mechanical vectors of scores of human and animal diseases.

## Sex determination in house flies

### Conserved dipteran sex determination pathway

**Figure 1:**
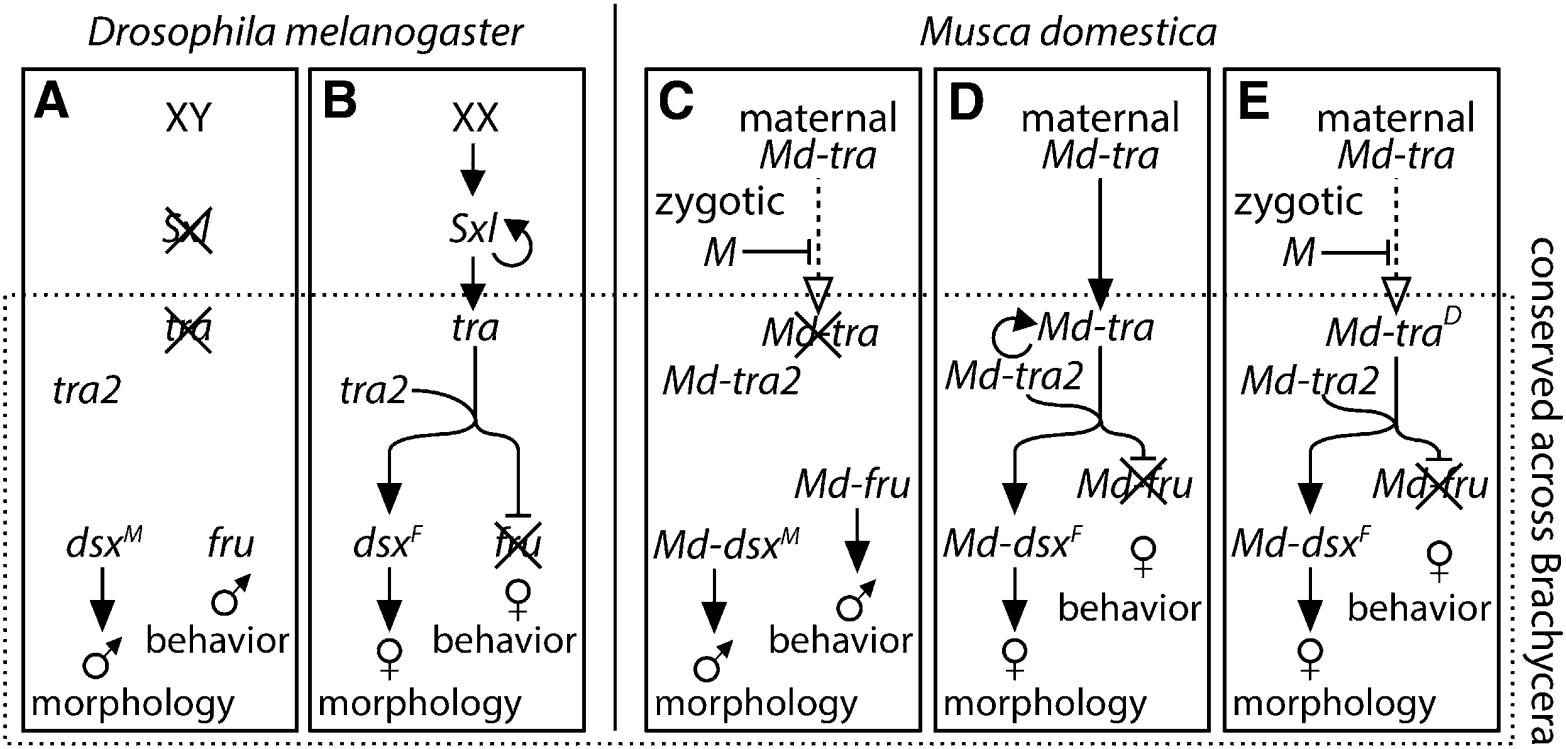
Sex-determination pathways. The (A) male and (B) female *Drosophila* sex-determination pathways are shown, along with the house fly (C) male-determining pathway, (D) canonical female-determining pathway, and (E) female-determining pathway via the action of *Md-tra^D^*. The core of the pathway that is conserved across brachyceran flies is contained within the dashed box. Abbreviations are described in the main text.

Sex determination in dipterans relies heavily on the differential regulation of alternative splicing between the sexes of genes expressed in both males and females (Salz 2011). The dipteran sex determination pathway (at least in Brachycera, or “higher” dipterans) consists of a core series of regulatory steps that are conserved in all brachyceran species examined thus far (Pomiankowski *et al.* 2004; Bopp *et al.* 2014; Geuverink and Beukeboom 2014) (Figure 1). At the start of this core pathway, the splicing regulator *transformer* (*tra*) is itself alternatively spliced to produce a functional transcript capable of encoding a full-length protein in females and a nonfunctional transcript with a premature stop codon in males (Boggs *et al.* 1987; McKeown *et al.* 1987; Pane *et al.* 2002, 2005; Lagos *et al.* 2007; Ruiz *et al.* 2007; Concha and Scott 2009). The factor responsible for the decision whether to produce the male or female splice form of *tra*, however, varies across species, as described in the next section.

Functional TRA protein in females, along with the product of the constitutively expressed *transformer 2* (*tra2*), promotes the splicing of the *Dmrt* homolog *doublesex* (*dsx*) into its female-specific isoform (*dsx^F^*), initiating female morphological development (Hoshijima *et al.* 1991) (Figure 1). TRA also causes the male-specific behavioral regulator *fruitless* (*fru*) to be spliced into a nonfunctional isoform in females (Ito *et al.* 1996; Ryner *et al.* 1996; Demir and Dickson 2005; Meier *et al.* 2013). The absence of functional TRA in males leads to male-specific splicing of *dsx* (*dsm^M^*) and splicing of *fru* into its functional male-specific isoform, initiating the development of male morphology and behavior, respectively.

### Variation in sex determination across dipterans

Although the aforementioned core sex determination pathway is conserved among dipterans, there is variation across species in how the pathway is initiated (Bopp *et al.* 2014). This is consistent with a model whereby sex determination pathways evolve by the change or addition of upstream components, because changes at the top of pathways are less likely to have deleterious effects (Wilkins 1995; Marin and Baker 1998). In the well-studied *Drosophila* system, *tra* splicing ultimately is controlled by the number of X chromosomes in the zygote (Bridges 1921; Pomiankowski *et al.* 2004; Erickson and Quintero 2007; Salz and Erickson 2010). Female zygotes (XX) have greater expression of X-linked “numerator” genes than male zygotes (XY). Two doses of the X-linked numerators leads to the expression of functional *Sex lethal* (*Sxl)* transcripts in females (Cline 1988; Duffy and Gergen 1991; Sefton *et al.* 2000), and the SXL protein autoregulates the continued splicing of *Sxl* into a functional transcript in females (Cline 1984) (Figure 1B). Functional SXL in females promotes splicing of *tra* into a functional isoform, whereas lack of functional SXL in males leads to nonfunctional splicing of *tra* (Valcárcel *et al.* 1993) (Figure 1A). *Sxl* is expressed equally in both sexes in other dipterans and is not a master regulator of sex determination in non-*Drosophila* species (Marin and Baker 1998; Schütt and Nothiger 2000; Saccone *et al.* 2002; Shearman 2002).

Other mechanisms of initiating the sex determination pathway in dipterans include environmental sex determination (*i.e.*, *Aedes stimulans*) (Horsfall and Anderson 1963), female-determining factors (*i.e.*, *M. domestica* and *Tephritidae*), or maternal genotype (*i.e.*, *Sciara* and *Chrysomya*) (Marin and Baker 1998; Saccone *et al.* 2002). Many dipteran species, including house fly, have a dominant male-determining factor (M) that is thought to inhibit the splicing of *tra* into a functional isoform in developing male zygotes (Traut and Willhoeft 1990; Dübendorfer *et al.* 2002; Bopp *et al.* 2014) (Figure 1C). In house flies, the homolog of *tra* (*Md-tra*) is expressed in the maternal germline, and lack of M in female zygotes allows maternal *Md-tra* to feed forward into zygotic expression of functional *Md-tra* (Hilfiker-Kleiner *et al.* 1994; Dübendorfer and Hediger 1998; Bopp 2010; Hediger *et al.* 2010) (Figure 1D). Zygotic TRA, along with TRA2, autoregulates the continued splicing of *Md-tra* into a functional isoform in the female zygote, whereas M breaks the feed-forward regulation of *Md-tra* in male zygotes (Figure 1, C and D) (Bopp 2010).

The ancestral brachyceran sex determination mechanism is hypothesized to be a male-determining gene located on the heteromorphic Y chromosome or one of the homomorphic chromosomes (Saccone *et al.* 2002; Vicoso and Bachtrog 2013). However, the position of M is not static in some species. In *Megaselia scalaris* the male-determining factor can be located on the first, second, or third chromosome (Traut 1994), although the transposing nature of the *M. scalaris* male-determining factor recently has been called into question (Hoehn and Noor 2015). In the mosquito *Culex tritaeniorhynchus* sex is determined by a male factor located on either linkage group I or III depending on the population (Baker and Sakai 1976). Variation in the linkage of M in house flies is detailed below.

The aforementioned developmental pathway regulates sex determination in somatic tissues. Germline sex determination in dipterans relies upon input from the somatic pathway, but the interdependence of the germline and somatic sex determination pathways is taxon-specific. For example, the *Drosophila* germline sex determination pathway combines information from the germline genotype with signals from the surrounding soma to determine the sex-specific developmental fate of germline tissues (Defalco *et al.* 2008; Casper and Van Doren 2009). Germline sex determination in house flies, on the other hand, depends entirely on the genetic sex of the surrounding soma (Hilfiker-Kleiner *et al.* 1994).

### *M. domestica*: M and F factors

Since the early report by Stevens (1908), many investigators have confirmed that the diploid chromosome number in the standard house fly, *M. domestica* L., is 12 consisting of 5 pairs of autosomes and a pair of sex chromosomes, and that the male is the heterogametic sex; that is, XX-type for the female and XY-type for the male (Hiroyoshi *et al.* 1982). The current nomenclature system (Wagoner 1969a) numbers house fly autosomes in order of decreasing length (*i.e.*, autosome I is longest and autosome V is shortest). These “standard” populations are composed of XY^M^ males and XX females. In these populations, maleness is determined by a Y chromosome that harbors a male-determining M factor (Y^M^) (Hiroyoshi 1964; Dübendorfer 2001). Two Y chromosome regions with male-determining activity (*i.e.*, M) have been identified that are functionally equivalent but nonredundant (Hediger *et al.* 1998). Both Y-linked male-determining regions are required for male development in the absence of autosomal M (A^M^) or X-linked M (X^M^) factors (Hediger *et al.* 1998).

The house fly X and Y chromosomes are largely heterochromatic and lack any known genes aside from M (usually on Y, but occasionally on X, see Table 1) (Bull 1983; Inoue and Hiroyoshi 1986). In addition, the number of Xs or Ys in a karyotype can vary up (*i.e.*, XXY or XXX) or down (*i.e.*, XO, OY) without any effect, as long as one X or Y is present (Bull 1983). Flies carrying only the short arm of the Y chromosome are also viable, but the long arm of the Y is not sufficient for viability in the absence of an X chromosome (Hediger *et al.* 1998). This suggests that any essential genes on the sex chromosomes must be located on the short arm of the Y (which also has a euchromatic segment) and on the homologous segment of the X chromosome. Almost all other muscid flies have five pairs of euchromatic chromosomes similar to the house fly “autosomes,” but not all species have the heterochromatic pair (Boyes *et al.* 1964; Bull 1983). However, nearly all species examined in other closely related families have five autosomes and a pair of sex chromosomes (Boyes and Van Brink 1965), suggesting that five autosomes plus the sex chromosomes (2n = 12) is the ancestral karyotype among most calyptrate flies, including muscids.

**Table 1:** Percentages*^a^* of M and *Md-tra^D^* in field collected strains of house fly

Location			I^M^	II^M^	III^M^	IV^M^	V^M^	Y^M^*^b^*	M/+*^c^*	M/M*^d^*	*Md-tra^D^**^e^*	Reference
Africa	S. Africa	Johannesburg-Pretoria area SA1	0	0	100		0	0		✓	✓	(Denholm *et al.* 1990)
“	S. Africa	Johannesburg-Pretoria area SA2	0	0	85		0	7.4	7.4	45	✓	“
“	S. Africa	Zinkwazi Beach	0	✓	✓	0	0	0			29	(Feldmeyer *et al.* 2008)
“	S. Africa	Umhlali	✓	✓	✓	0	✓	0			79	“
“	S. Africa	Hammarsdale	0	✓	✓	0	0	0			92	“
“	S. Africa	Ashburton	✓	✓	0	0	✓	0			13	“
“	S. Africa	Mooi River	0	✓	✓	0	0	0			29	“
“	S. Africa	Warden	0	0	70	0	0	30	✓		15	“
“	S. Africa	South Africa combined								26		“
“	Tanzania	Same	0	100	0	0	0	0			100	“
“	Tanzania	Moshi	0	100	0	0	0	0			100	“
“	Tanzania	Makuiuny	0	80	0	0	0	20	✓		100	“
“	Tanzania	Arusha	0	100	0	0	0	0			100	“
“	Tanzania	Karatu	0	80	0	0	0	20	✓		85	“
“		Tanzania combined								62		“
“	Tanzania										✓	(Scott *et al.* 2014a)
Australia	Australia	Ipswich	0	44	70	2	0	7	92	70	✓	(Hamm and Scott 2009)
“	“	Bowhill	0	✓	✓	0	✓				✓	(Wagoner 1969b)
Asia	Japan	Furano	0	0	9	0	0	91	0		0	(Tomita and Wada 1989b)
“	“	Sapporo	0	0	29	0	0.6	70	0.3		0.6	“
“	“	Akkeshi	0	0	21	0	0	79	0		0	“
“	“	Obihiro	0	0	12	0	0	88	0		0	“
“	“	Hachinohe	0	0	38	0	0.5	57	2		4	“
“	“	Niharu	5	0	32	0	0	64	0			“
“	“	Togakushi	0	8	58	0	0	35	4		28	“
“	“	Haga	0	2	96	0	0	2	4		17	“
“	“	Miyagi	0	3	35	0	0	63	5		0	“
“	“	Hokota	0	2	57	0	0	40	5			“
“	“	Kofu	0	0	70	0	0	30	24		29	“
“	“	Yumenoshima	0	1	74	0	0	25	68	48	99	“
“	“	Aio	2	31	29	3	22	12	2		1	“
“	“	Kasuya	1	16	39	0	18	26	20		38	“
“	“	Nangoku	3	0	24	0	3	70	6		13	“
“	“	Haruno	0	0	34	0	0	66	2		0	“
“	“	Hachijo	0	0	100	0	0	0	0		0	“
“	“	Okinawa	4	41	48	2	0	4	15		47	“
“	“	Ishigaki	0	32	54	0	0	14	2		4	“
“	“	Kirishima			0							(Hiroyoshi 1964)
“	“	Nichinan			0							“
“	“	Sakurai			0							“
“	“	Kitakyushu	✓	✓	✓	0	0	✓*	✓	0		(Tsukamoto *et al.* 1980)
“	“	Kitakyushu	0	✓	✓	0	✓	✓*	✓	0		“
“	“	FR 83			80				0		0	(Tomita and Wada 1989a)
“	“	OH 83	0	0	21	0	0	0	0		0	“
“	“	AK 83	0	0	21	0	0	0	0		0	“
“	“	SP-YG 83	0	0	40	0	0	0	0		0	“
“	“	SP-YG 84	0	0	24	0	0	0	0		0	“
“	“	SP-OD 84	0	0	33	0	0	0	0		0	“
“	“	IK-RS 84	0	0	24	0	0	0	2		3	“
“	“	IK-YU 84	0	0	30	0	0	0	0		0	“
“	“	IK-BN 84	0	0	35	0	0	0	0		0	“
“	“	OT-ZB 84	0	0	29	0	0	0	0		0	“
		Osaka									✓	“
Asia/Europe	Turkey	Giresun			✓						✓	(Cakir 1999)
“	“	Ordu			✓						✓	“
“	“	Trabzon					✓				✓	“
“	“	Giresun		0	✓	0			✓			(Cakir and Kence 2000)
“	“	Trabzon		0	0	✓			✓			“
“	“	Kayrak		0	✓	0						“
“	“	Simav		0	✓	0						“
“	“	Izmit		✓	✓	0			✓			“
“	“	Iskenderun		✓	✓	✓			✓			“
“	“	Balikesirr		0	✓	0						“
“	“	Polatli		0	0	0						“
		Trabzon									✓	(Scott *et al.* 2014a)
Europe	British Isles	Fm31	0	0	0		0	0*		✓	✓	(Denholm *et al.* 1985)
“	“	Fm39	0	0	✓		0	✓ *			✓	“
“	“	Fm42	0	0	0		0	✓				“
“	“	Harpenden							✓	✓		
“	“	Fm44	0	0	✓		0	✓	25	35-52		“
“	“	Fm45	0	0	✓		0	✓			✓	“
“	England	Fm6	0	0	6	0	0	94*	6	✓		(Denholm *et al.* 1983)
“	“	Fm22	0	0	2.9	0	0	69*	29	✓		“
“	Italy	12 populations	0	0	0	0	0	✓				(Franco *et al.* 1982)
“	“	11 populations		✓	✓					✓	✓	“
“	“	IT1	0	0	12	0	0	52			44	(Kozielska *et al.* 2008)
“	“	IT2	0	25	9	0	0	44			43	“
“	“	IT3	0	0	0	0	0	50			10	“
“	“	IT4	12	9	45	0	0	42	✓		100	“
“	“	IT5	2	17	50	0	9	62	✓		100	“
“	“	IT6	3	13	32	0	0	68	✓		95	“
“	“	IT7	0	3	53	0	0	17			78	“
“	“	IT8	9	3	86	3	3	16	✓		100	“
“	“	IT9	8	17	46	0	0	6			86	“
“	“	IT10	3	0	55	3	0	0			95	“
“	“	IT11	0	0	76	0	0	3			96	“
“	“	IT12	0	0	56	0	0	8			47	“
“	Switzerland	Switzerland	0	0	0	0	0	50			5	“
“	Germany	GE1	0	0	0	0	0	50			0	“
“	“	GE2	0	0	0	0	0	50			0	“
	France	Faverges									✓	(Scott *et al.* 2014a)
	Spain	Santa Fé									✓	“
N. America	USA	Texas			10	0				✓		(McDonald and Overland 1974)
“	“	North Dakota			8	0				✓		“
“	“	Florida			100	0		0				“
“	“	Florida	0	0	100	0	0	0				(Hamm *et al.* 2005)
“	“	North Carolina 2002	0	0	20	0	0	78	2.4	0		“
“	“	North Carolina 2006	0	0	19	0	0	78	1.4	1.4		(Hamm and Scott 2008)
“	“	North Carolina 2007	0	0	2.3	0	0	95	0	2.3	4.2	“
“	“	New York	0	0	4.4	0	0	96				(Hamm *et al.* 2005)
“	“	Maine	0	0	0	0	0	100				“
“	“	California- Chino	0	0	15	0	0	85	✓	✓		(T. Shono and J. G. Scott, personal communication)

Blank cells indicate no information available (*i.e.*, experiments not conducted or marker strain for specific autosome not used). ✓, detected, but not quantified.

a Values can vary from one study to another primarily based on how males with multiple M factors were categorized. See the individual papers for details.

b Y^M^ values from some studies indicate only that M was not linked to an autosome, thus linkage of M to Y or to X are possible in some of these studies.

c Percentage of males being heterozygous for M at more than one linkage group (*e.g.*, II^M^/II; III^M^/III or III^M^/III; XY^M^). Zeroes indicate that appropriate methods for detection were used and that none were found.

d Percentage of males producing only male offspring (*i.e.*, homozygous for at least one autosome (A^M^/A^M^, X^M^/Y^M^, or X^M^/X^M^). Zeroes indicate that appropriate methods for detection were used and that none were found.

e Populations that have homozygous M males can be reasonably assumed to have *Md-tra^D^* females. However, these cells were left blank unless there was detection (✓) or quantification of *Md-tra^D^*.

* X^M^ males were found most commonly in this population (male determining factor did not map to an autosome and a male had a karyotype of XX).

It was shown recently that the gray flesh fly, *Sarcophaga bullata*, X chromosome is homologous to the *Drosophila* “dot” chromosome (chromosome 4 in *D. melanogaster*), and this chromosome is likely to be the ancestral X chromosome of Brachycera (Vicoso and Bachtrog 2013). The house fly sex chromosomes, therefore, likely reflect an ancient X/Y pair, and decreases in chromosome number among the Muscidae are likely the result of fusions of the ancestral sex chromosomes with one of the five autosomes.

“Autosomal” (A^M^) or “atypical” (Rubini *et al.* 1980) house fly strains have the M factor located on one or more of the five autosomes (I−V) or the X (Note: The A^M^ designation is a bit misleading because X^M^ males also are considered “atypical”). The M factor has been shown to have varying degrees of strength depending on its location (Schmidt *et al.* 1997b). I^M^ males are weak intersexes expressing female-specific yolk proteins (Schmidt *et al.* 1997b) and both the male and female isoforms of *Md-dsx* (Siegenthaler *et al.* 2009). The suggested cause of this is the presence of prominent stretches of heterochromatin on autosome I (Hediger *et al.* 1998; Dübendorfer 2001). Y^M^ (if multiple copies of M are present), III^M^, and V^M^ show strong male-determining effects in the soma and impede the activity of *Md-tra* when introduced into the female germline by transplantation of progenitor germline cells (Schmidt *et al.* 1997b).

It is hypothesized that A^M^ or X^M^ factors are the result of transposition of the Y-linked M (Hiroyoshi 1964), and several lines of evidence suggest that M “inserts” into a single location on each autosome or X (Inoue and Hiroyoshi 1986). House flies in South East England contained a high frequency of X^M^ individuals and “a small secondary constriction on X appeared to indicate reliably the presence of X^M^” (Denholm *et al.* 1983). The linkage of M was investigated using three I^M^ strains and two III^M^ strains collected in Japan (Inoue *et al.* 1983). All three I^M^ factors mapped to the right of the *black puparium* (*bp*) gene, and M was found tightly linked to *pointed wings* (*pw*) in both III^M^ strains, suggesting that M occupies a definite site on the respective chromosomes (Inoue *et al.* 1983). The authors concluded that A^M^ factors are located in centric heterochromatin on each autosome and M factors on a given chromosome are all at the same locus. Alternatively, it was proposed that the M factors on the different autosomes are different genes that adopt the function of male-determiner through mutation (Bopp 2010).

*Md-tra* is located on autosome IV and has two different functional variants. The “wild-type” allele is sensitive to inhibition by M, whereas a dominant allele (*Md-tra^D^*, formerly *F^D^*) is resistant to M and acts as a female-determining factor (McDonald *et al.* 1978; Inoue and Hiroyoshi 1986; Cakir 1999; Hediger *et al.* 2010) (Figure 1E). The *Md-tra^D^* allele may have invaded natural populations because of sex ratio selection (Hamilton 1967; Bull and Charnov 1977; Kozielska *et al.* 2006). Populations that contain males with multiple M factors (III^M^ and Y^M^, for example) or males homozygous for an A^M^ can skew the sex ratio away from 1:1 male:female. The presence of *Md-tra^D^* in the zygotic genotype causes female development even in the presence of up to three M factors (McDonald *et al.* 1978; Schmidt *et al.* 1997b; Hediger *et al.* 1998), potentially balancing the sex ratio in populations with multiple M factors. In populations in which males are exclusively A^M^/A^M^ and the *Md-tra^D^* allele segregates (Franco *et al.* 1982; Denholm *et al.* 1983, 1985; Denholm *et al.* 1990), females are the heterogametic sex (*Md-tra^D^*/*Md-tra^+^*). *Md-tra^D^* has been reported in populations from Africa, Asia, Australia, Europe, and North America (Table 1).

**Figure 2:**
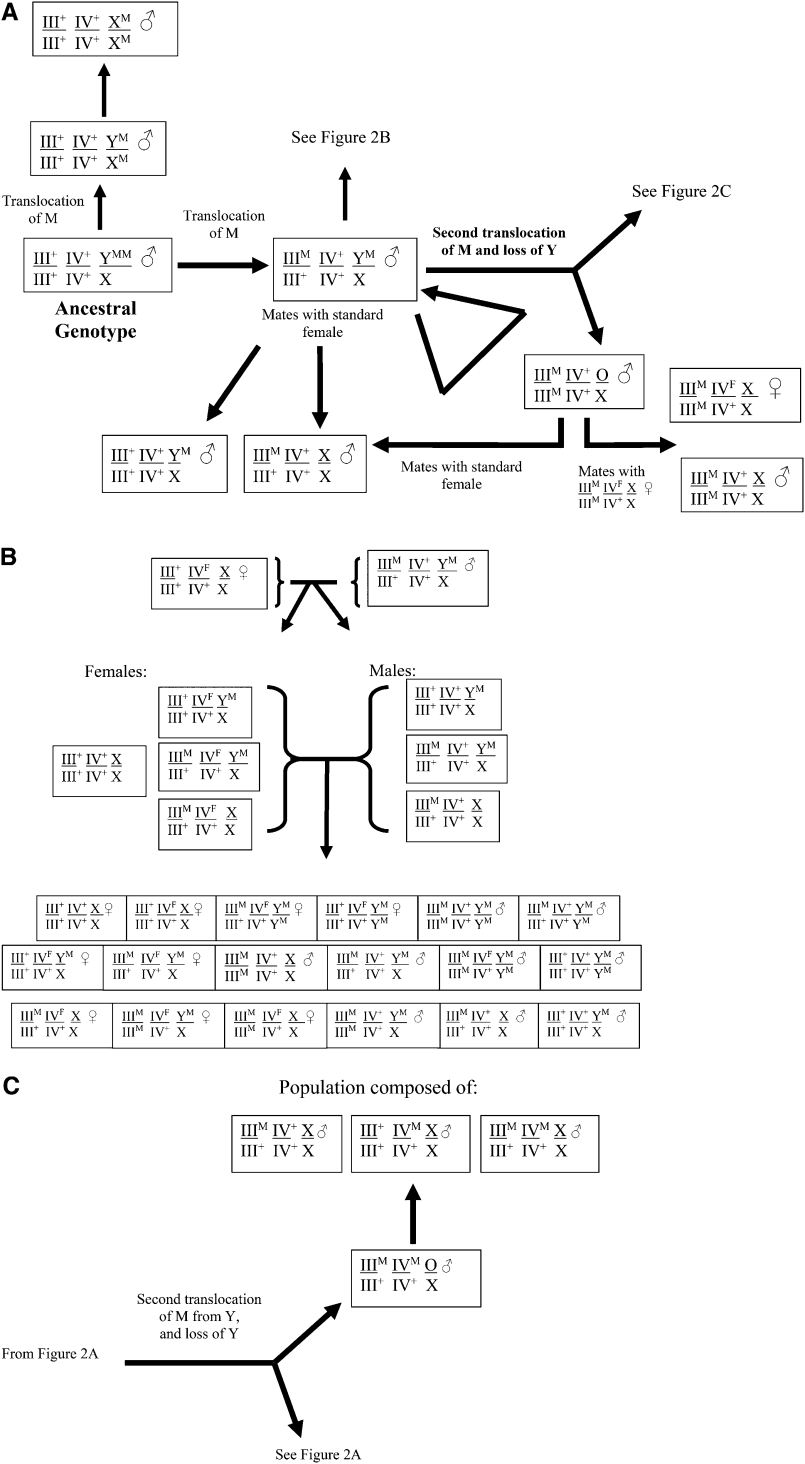
Schematic representation of the evolution of changes in the linkage of M and frequency of F (*Md-tra^D^*) in the house fly, *M. domestica*. Autosomes III and IV are used for illustration purposes but could be any of the autosomes (see Table 1). Genotype is given only for the male unless otherwise specified. Females are assumed to be III^+^/III^+^ ; IV^+^/IV^+^; XX unless otherwise specified. (A) Changes possible from the ancestral state (XY^M^, with no autosomomal males). (B) Continued from (A). Schematic representation of the evolution of males and females homozygous for M. (C) Continued from (A). Schematic representation of the evolution of males with copies of M on different autosomes. The A^M^ factors are assumed to be derived from the M factor on Y (Hiroyoshi 1964), and the M factors are thought to incorporate into a specific site on each autosome (Inoue *et al.* 1983).

Figure 2 presents a hypothetical general scheme of the changes that are likely to occur as a house fly population evolves from one type of sex determination system to one of the others. The scheme represented in Figure 2 assumes that M can be mobilized from Y to another chromosome (once or twice) with the resulting loss (over time) of the Y chromosome. The proposed scheme accounts for genotypes found in nature, although some karyotypes that rarely have been detected (Table 2) are not included for the sake of simplicity.

**Table 2:** Percentage of male house flies with specific karyotypes

				Percentage							
Location			n	XY	XX	XO	OY	XXX	XXY	YY	Reference
Africa	S. Africa	SA1	31		90	10					(Denholm *et al.* 1990)
“	“	SA2	33	30	64					6	“
Asia/Europe	Turkey	Antalya	30	53	47						(Cakir and Kence 1996)
“	“	Incekum	31	74	26						“
“	“	Anamur	31	77	23						“
“	“	Gulnar	32	34	66						“
“	“	Kayrak	30	10	90						“
“	“	Y. Cadiri	32	41	59						“
“	“	Silifke	30	30	70						“
“	“	Atakent	30	60	40						“
“	“	Mersin	36	42	58						“
“	“	Adana	30	43	57						“
“	“	Yumurtalik	30	23	77						“
“	“	Karatas	25	56	44						“
“	“	Ceyhan	30	70	30						“
“	“	Samsun	32	75	25						“
“	“	Giresun	30	0	100						“
“	“	Trabzon	33	0	100						“
“	“	Rize	30	13	87						“
“	“	Artvin	33	24	76						“
“	“	Erzurum	32	53	47						“
“	“	Erzincan	36	83	17						“
“	“	Sivas	33	91	9						“
“	“	Yozgat	30	77	23						“
“	“	Izmit	30		100						“
“	“	Isparta	30	40	60						“
“	“	Bursa	31	68	32						“
“	“	Tokat	36	69					31		“
“	“	Istanbul	30	23	77						“
“	“	Iskenderun	29	24	76						“
“	“	Afyon	28	61	39						“
“	“	Usak	32	50	50						“
“	“	Ismir	31	29	71						“
“	“	Manisa	29	55	45						“
“	“	Balikesir	28	25	75						“
“	“	Simav	29	30	70						“
“	“	Ankara	31	71	29						“
“	“	Polatli	31	97	3						“
Europe	UK	Fm 42	51	88			4		8		(Denholm *et al.* 1985)
“	“	Fm 39	33	58	39	3					“
“	“	Fm 31	28	21	79						“
“	“	Fm 44	47		98			2			“
“	“	Fm 45	48	75	19		4		2		“
“	“	Harpenden	223	5	93	2		0			“
“	“	Fm 3	19	21	79						(Denholm *et al.* 1983)
“	“	Fm 9	33		100						“
“	“	Fm 6	36		89	11					“
“	“	Fm 13	29		100						“
“	“	Fm 11	11		100						“
“	“	Fm 14	27	11	89						“
“	“	Fm 22	46	2	98						“
“	“	Fm 29	22	14	82			5			“
“	France	M1	87	77	15					8	(Franco *et al.* 1982)
“	France	M2	49	59	41						“
“	Yugoslavia	M3	69	46	52					1	“
“	Italy	M4	92	85						15	“
“	Italy	M5 (2r)	178	88						12	“
“	Italy	M5 (2r)	94	67	17					16	“
“	Italy	M6	44	50	50						“
“	Italy	M7 (2r)	149	64	36					1	“
“	Italy	M8	56	84	7					9	“
“	Italy	M9 (2)	52	83	15					2	“
“	Italy	M10	72	31	67					3	“
“	Italy	M11	46	4	96						“
“	Italy	M12	61	39	57					3	“
“	Italy	M13	63	2	92					6	“
“	Italy	M14	72	19	81						“
“	Italy	M15	43	56	44						“
“	Italy	M16	54	35	61					4	“
“	Italy	M17	62	2	98						“
“	Italy	M18	25	4	96						“
“	Italy	M19	40	15	85						“
“	Sardinia	M20	96	10	90						“
“	Sardinia	M21	68	9	91						“
“	Iceland	S1	30	100							“
“	Denmark	S2-3-42	105	100							“
“	Netherlands	S5-6^r^-7	162	100							“
“	Germany	S8	85	100							
“	Switzerland	S9-10-11	167	100							“
“	Italy	A1-2-3-4-5	130		100						“
“	Italy	A6-7-8-9	253		100						“
“	Sicily	A10-11	83		100						“
		Total	4416	45	53	0.27	0.09	0.07	0.36	1.8	

Blank cells equal 0%.

In addition to the variation in M and *Md-tra^D^* observed in natural populations, other alleles of both genes have been isolated in the laboratory. A loss-of-function mutation in *Md-tra*, *Md-tra^man^* (formerly *F^man^*), turns wild-type *Md-tra* into a female-determining allele in the absence of M (Schmidt *et al.* 1997a). The *Md-tra^man^* mutation removes TRA/TRA2 binding sites, which likely prevents TRA from autoregulating the splicing of the *Md-tra^man^* allele into a functional female-specific isoform (Hediger *et al.* 2010). In laboratory strains carrying the *Md-tra^man^* mutation and lacking any M factors, males were the homogametic sex (homozygous for *Md-tra^man^*) and females were heterogametic (*Md-tra^man^*/*Md-tra*^+^) (Schmidt *et al.* 1997a).

The activity of *Md-tra* can be inhibited by mutations that affect the maternal germline or zygote separately, which can convert the house fly sex determination system into one controlled by the maternal genotype. A recessive mutation on chromosome IV, which is likely a hypomorphic allele of *Md-tra* that is lacking maternal germline function (Schmidt *et al.* 1997a), was fortuitously named *transformer* (*tra*) by Inoue and Hiroyoshi (1986). Homozygous females (*tra/tra*) produced intersexes or males without an M factor, whereas heterozygotes (*tra*/+) produced mostly females when mated to males lacking M (Inoue and Hiroyoshi 1986). Zygotes carrying an M factor developed into males regardless of whether the mother had one or two copies of this *tra* mutation (Inoue and Hiroyoshi 1986). The house fly *tra* mutation produces a situation reminiscent of that observed in naturally occurring systems in which maternal genotype determines sex, such as *Chrysomya rufifacies*, suggesting a mutational mechanism by which maternal effect sex determination can evolve (Scott *et al.* 2014b).

Similarly, the germline and zygotic activity of M can be separated, also creating house fly strains with maternal effect sex determination. The *Arrhenogenic* (*Ag*) mutation (Vanossi and Rovati 1982) on the first chromosome was most likely a hypomorphic allele in the promoter region of an M locus on chromosome I that maintained germline expression but impaired expression in the zygote (Schmidt *et al.* 1997a; Dübendorfer *et al.* 2002). Females that were heterozygous for the *Ag* allele failed to activate *Md-tra* in the germline (because the germline activity of M inhibits *Md-tra*) and produced all male offspring (Hediger *et al.* 2010). When this strain was maintained in the laboratory, homozygous wild-type females produced all-female offspring because males of this strain lack an M allele with zygotic activity (Hediger *et al.* 2004).

Early studies revealed a virtual lack of crossing-over in male house flies (McDonald 1971; Lester *et al.* 1979), consistent with what is observed in most other dipterans (White 1973; Gethmann 1988). This facilitated genetic studies to determine the chromosomal locations of sex-determining factors. Later worked revealed that the crossover frequencies in males vary, depending on the genes examined and the populations used. Reported values range between 0–0.53% (Hamm *et al.* 2005; Hamm 2008), 0.03–0.11% (Sullivan 1961), 9.3–31% (Lester *et al.* 1979), and 7–28% (Feldmeyer *et al.* 2010). Intriguingly, greater male recombination rates tend to be associated with A^M^ (Sullivan 1961; Hiroyoshi *et al.* 1982; Inoue and Hiroyoshi 1982; Gethmann 1988; Hamm *et al.* 2005; Hamm 2008; Feldmeyer *et al.* 2010).

Evidence for male recombination in laboratory experiments could be the result of meiotic crossing over and/or premeiotic events such as mitotic recombination. Genomic rearrangements, Y-autosome translocations, mobile element insertions, and transposable M factors increase the frequency of male recombination in multiple different dipteran species, but this is not necessarily because of an increased rate of meiotic recombination (Gethmann 1988). Asymmetrical reciprocal recombinant classes suggest that many examples of male recombination in house fly might be the result of premeiotic events (*e.g.*, mitotic recombination) or aneuploid segregants, not meiotic recombination (Rubini *et al.* 1980; Gethmann 1988). *Megaselia scalaris* also has a transposing male-determining factor, and male recombination in this species appears to result from premeiotic events (Gethmann 1988). A nonrandom association between transposition of the *M. scalaris* M and male recombination suggest that the two processes may be caused by similar underlying factors in the male germline (Mainx 1964). This parallels the association between transposable element derepression and male recombination observed in *Drosophila*, suggesting a common effect of transposable elements and transposing M factors on genome instability in the premeiotic male germline (Gethmann 1988). Alternatively, elevated male recombination in A^M^ genotypes might reflect the early stages of differentiation in a nascent sex chromosome system where male recombination is not yet repressed (Feldmeyer *et al.* 2010). Additional experiments are needed to test these hypotheses.

### Geographic distribution of A^M^ *vs.* Y^M^ and/or X^M^ males

**Figure 3:**
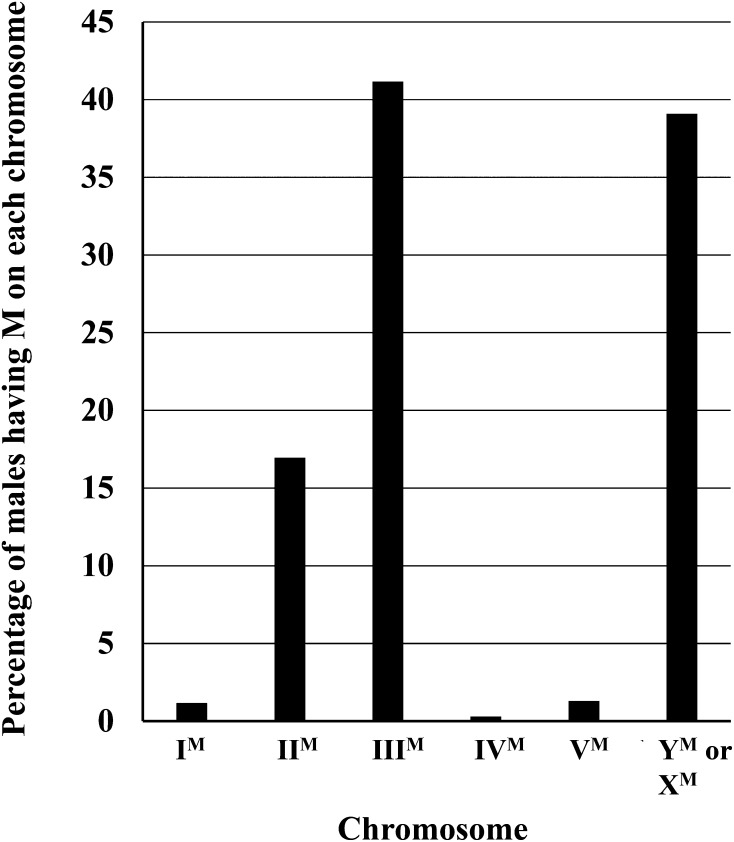
The relative percentage of males with M on each of the chromosomes. Results were calculated from the data in Table 1. Values represent relative percentages, as different reports used in Table 1 accounted for males with multiple M factors using different calculations. Studies failing to find a linkage of M to an autosome called these strains Y^M^, although in the absence of karyotype information these strains could also be X^M^.

Non-Y^M^ house fly populations exist in nature throughout the world, and M factors can be found at a wide range of frequencies. A summary of the papers reporting on the linkage of M is given in Table 1. A^M^ males have been found on most continents, with the notable exception of Central and South America, for which there have been no published studies. M has been found most frequently on autosome III, followed by the Y or X chromosome, and then autosome II (Figure 3). M is rarely found on autosomes I, IV, or V (Figure 3). Most studies that do not find M on an autosome assume that this is a Y^M^ strain, based on the belief this is the ancestral condition. However, M may also be X-linked, so without karyotyping, distinguishing between Y^M^ and X^M^ is not possible. A summary of the different karyotypes found for male house flies (Table 2) reveals the frequencies of Y^M^ and X^M^ are about equal across the populations surveyed. Other male karyotypes (*e.g.*, XO, OY, XXX, XXY, YY) were detected (Table 2), but were overall rare (<2%). There are also many populations that contain males with multiple M factors. The Ipswich (Australia) population has the highest number of multiple M males and homozygous M males found to date—92 and 70%, respectively (Hamm and Scott 2009). This represents an extreme case and one in which the population appears headed for females to become the heterogametic sex. The presence of *Md-tra^D^* was confirmed in this population, although the frequency of *Md-tra^D^* was not determined (Hamm and Scott 2009).

Like A^M^ males, females with *Md-tra^D^* have also been found throughout the world (Table 1). The frequency of *Md-tra^D^* in females varies from 0% in some populations to 100% of the females in four locations in Tanzania. It would be expected that all males would either carry multiple M factors or be homozygous for M in populations where *Md-tra^D^* is found in all females, but unfortunately this was not investigated in these studies. One *Md-tra^D^* haplotype (accession# GU070694) contains three small intronic insertions/deletions (indels), a small insertion in a male-specific exon, and one nonsynonymous substitution in the coding region (Hediger *et al.* 2010). The indels are thought to allow for the zygotic splicing of the *Md-tra^D^* allele into a functional isoform in the absence of the feed-forward activity of *Md-tra* from the maternal germline, and they may prevent the negative regulation of M (Hediger *et al.* 2010) (Figure 1E). The same *Md-tra^D^* haplotype was found in seven different populations sampled across Europe, North America, Asia, Africa, and Australia (Scott *et al.* 2014a). In contrast, multiple *Md-tra*^+^ haplotypes were found in these populations, leading to speculation that *Md-tra^D^* may have a single evolutionary origin followed by a recent global spread.

Surveys of house flies on multiple continents (in the northern hemisphere) have revealed latitudinal gradients of A^M^ and Y^M^ populations, with Y^M^ males most common in the north and A^M^ or X^M^ more common in the south. For example, in European populations ranging from Sicily to Denmark and Iceland, Franco *et al.* (1982) found A^M^ and X^M^ males more often below the 44^th^ parallel and Y^M^ males more frequent in the north. McDonald *et al.* (1975) reported latitudinal variation in A^M^ in North America, with populations from North Dakota, Texas, and Florida containing III^M^ males at 0.8%, 10.4%, and 100%, respectively. However, this study did not survey M factors on other autosomes and found a low frequency of A^M^ males in Texas, a southern location. Stronger evidence for a North American latitudinal gradient in A^M^ was revealed in a survey that sampled from Florida (29° 41′ latitude) to Maine (44° 2′) (Hamm *et al.* 2005). In Florida, 100% of the males possess the M factor on chromosome III. North Carolina had 20% III^M^ and 2.35% with both Y^M^ and III^M^ in the same individual. Fewer III^M^ males were located in New York (4.35%), and the Maine population was entirely XY^M^ or XX^M^. This range in latitude was similar to that in Japan, where a north-south cline was also observed (Tomita and Wada 1989b). *Md-tra^D^* is distributed sporadically throughout the Japanese populations at frequencies ranging between 0 and 99% of females (Tomita and Wada 1989b).

Other patterns have been observed in the spatial distribution of A^M^. For example, a radial cline was detected in the British Isles (Denholm *et al.* 1985). Populations in central England were predominantly X^M^X^M^, whereas XY^M^ males were found to inhabit the north. The frequency of *Md-tra^D^*, X^M^, and a rarer III^M^ decreased on moving north, east, and west. Karyotype data revealed Y^M^ to be extremely rare in most strains collected in the south of England, increasing in frequency upon moving north. The Y chromosome morphology appeared small in southeast England, and the longest Y chromosome was observed in Scotland (the north). Two sites at the same latitude differed in the frequency of the Y chromosome, supporting the radial cline hypothesis.

Franco *et al.* (1982) also reported an altitudinal gradient in Europe, with A^M^ populations less than 100 meters above sea level. A^M^ males decreased as the altitude increased. An altitudinal cline also was detected in Turkey. Cytological examinations revealed frequencies of XX males (assumed to be A^M^) ranging from 3.22 to 100% (Cakir and Kence 1996), and XX males were present in 10 of the 36 populations at frequencies greater than 70% (Table 2). There were fewer XX males in the central and eastern Anatolian highlands than in the coastal regions (Cakir and Kence 1996). The Y chromosome was absent in three populations (Izmit, Giresun, and Trabzon). Further research in Turkey established the existence of III^M^, V^M^, and *Md-tra^D^* (Cakir 1999) (Table 1).

Strains with different numbers of M factors have the ability to produce a variety of sex ratios depending on their genetic makeup (Table 3). According to Fisher’s theory, the equilibrium sex ratio is most likely to be 1:1 due to the notion that if one sex is rare, it will have greater reproductive success (Goodenough *et al.* 1993), and a modeling study in house fly supported this optimal ratio for house flies (Kozielska *et al.* 2006). The most common way to maintain equal sex ratios is for parents to have equal numbers of male and female offspring, and any deviation should be automatically corrected by selection in favor of the other sex (Fisher 1930; Hamilton 1967). In house flies, populations that contain only one M factor found only in a heterozygous state (either Y-linked, X-linked, or autosomal) will produce a 1:1 ratio of males to females. However, deviations from a 1:1 sex ratio can be obtained when a male carries multiple M factors (Table 3). If a normal female produces only sons, her mate must be homozygous for at least one A^M^ (or X^M^). This male may or may not have additional M factors. A male heterozygous for the M factor on two different chromosomes will produce 75% male offspring, whereas a male with five M factors in heterozygous form will produce 96.9% male offspring (Table 3). These situations all assume that the female does not carry *Md-tra^D^*. The house fly sex determination polymorphism therefore provides a mechanism by which biased sex ratios are produced in the absence of meiotic drive or some other non-Mendelian sex-ratio distortion system.

**Table 3:** Examples of the different percentages of males produced by different male genotypes assuming that the population lacks *Md-tra^D^*

Male Genotype	% males in F_1_
III^M^/III	50
XY^M^	50
II^M^/II;III^M^/III	75
III^M^/III;XY^M^	75
II^M^/II;III^M^/III;/IV^M^/IV	87.5
III^M^/III;IV^M^/IV;XY^M^	87.5
I^M^/I;II^M^/II;III^M^/III;IV^M^/IV	93.4
I^M^/I;II^M^/II;III^M^/III;IV^M^/IV;V^M^/V	96.9
II^M^/II;III^M^/III^M^;XY^M^	100
III^M^/III^M^	100
II^M^/II;III^M^/III^M^	100
II^M^/II^M^;III^M^/III^M^	100
II^M^/II;III^M^/III^M^;IV^M^/IV;XY^M^	100

Nearly all of these genotypes have been observed in field collected flies, although others exist as well (Hamm *et al.* 2005; Hamm and Scott 2008, 2009). In theory, any of the five autosomes could exhibit these genotypes and produce the same proportion of male offspring (*e.g.*, III^M^/III^M^ or V^M^/V^M^ both produce only male progeny in the absence of *Md-tra^D^*).

### Little evidence for changes in frequency of A^M^ males over time in field populations

Despite the variation that occurs between populations, studies on the relative frequency of A^M^ *vs.* Y^M^ over time within field populations, from the United States and Europe, have shown that the populations are relatively unchanged from the 1970s onwards. Male flies collected in 1973, 2003, and 2009 from Florida were 100% III^M^ (McDonald *et al.* 1975; Hamm *et al.* 2005; Kavi *et al.* 2014). The frequency of A^M^ males in Europe was evaluated in 2006 by the use of 15 collections from southern Italy to northern Germany and compared with collections made 25 years earlier. There was no clear change in the distribution of sex-determining factors (Kozielska *et al.* 2008). In flies from North Carolina, frequencies of III/III; XY^M^, III^M^/III; XX, and III^M^/III; XY^M^ males were unchanged (karyotypes were assumed, but not determined) between 2002 and 2006 (Hamm and Scott 2008). Field-collected flies from this population in 2007 showed a slight increase in the frequency of XY^M^ males and a slight decrease in the frequency of III^M^/III males (relative to 2002 and 2006), suggesting that the relative frequency of XY^M^ and III^M^/III can vary slightly over time (Hamm and Scott 2008). The first recorded autosomal male (III^M^) factor in the northeast United States was reported in 2003 (Shono and Scott 2003) from flies collected in New York (and laboratory selected with the insecticide spinosad). In contrast, field-collected flies from New York in 1980 (Scott *et al.* 1984) and 1987 (Konno and Scott 1991) (that were also selected with insecticides) were XY^M^ or XX^M^, leading to the suggestion that the frequency of A^M^ might be increasing (Shono and Scott 2003). A 2005 study showed that flies from New York were III^M^ at a frequency of 4.35% of the population (Hamm *et al.* 2005), so it is unclear whether the failure to detect A^M^ males in 1980 and 1987 was due to the low frequency of A^M^ or if the frequency is actually increasing.

In addition to field-collected strains, the linkage of M has been determined in several laboratory strains. These results are summarized in Supporting Information, Table S1. These data, particularly if the collection site is known, can provide additional information about the distribution of A^M^ males. However, colonization in the laboratory will likely alter the frequency of the different M factors (Hamm and Scott 2008). The frequency of the linkage of M in laboratory strains was similar to that found for field collections, with III^M^ and Y^M^ being the most common. Curiously, M in the SRS strain maintained by different laboratories has been linked to V (Hamm *et al.* 2005), Y (Milani *et al.* 1967; Franco *et al.* 1982), and III (Hamm 2008). It is difficult to assess whether these differences are attributable to local adaptation or separate contamination events.

### Studies of the relative fitness of A^M^ and Y^M^ males

Franco *et al.* (1982) noted that “all the papers concerning the karyotype of *Musca domestica* L. (2n = 12) published between 1908 and 1948 . . . reported the presence of *XX* females and *XY* males . . . It can be assumed that the authors, being European, examined houseflies of European origin. Since 1958, cases of sex-limited inheritance, interpreted *a posteriori* as due to autosomal sex-determinants, have been described in several strains of houseflies of non-European origin.” Starting in about 1960, the reports of A^M^ males increased, but it is not clear whether this was a result of the recent invasion of autosomal M factors, incomplete sampling in earlier studies, or neglecting to search for A^M^. This spread of A^M^ males (perceived or real) led to the suggestion that it might be causally related to selection for insecticide resistance (Hiroyoshi 1980), although later the author no longer held that opinion (personal communication to R. M. Sawicki, cited in Denholm *et al.* 1983).

Insecticide resistance in the house fly has been studied widely and is most commonly not sex-linked (Tsukamoto 1983), although there are some exceptions. One study found that the frequency of III^M^ males increased after selection with insecticide (permethrin), and the authors concluded this could be due to either tight linkage between the locus conferring resistance and the III^M^ locus or to genetic drift (Denholm *et al.* 1983). A study directly comparing insecticide resistance levels and frequency of A^M^ males in four geographically separate populations found no correlation between resistance (including *kdr-type* resistance on chromosome III) and the frequency of A^M^ (or III^M^) males (Hamm *et al.* 2005). Although there is an important mechanism of pyrethroid resistance on autosome III (*kdr-type*), this resistance is inherited as an incompletely recessive trait (*i.e.*, heterozygotes have only low levels of resistance) (Shono 1985). It is therefore unlikely that selection for pyrethroid resistance in heterozygotes drove the invasion of III^M^. However, there are two reports of sex-linked (male-limited) inheritance of insecticide resistance in natural populations (Kerr 1960; Kence and Kence 1992). The first was a report of about eightfold greater resistance to dichlorodiphenyltrichloroethane in males than females in the Canberra strain, but the linkage of resistance and M was not reported (Kerr 1960). The second was a report of greater levels of malathion resistance in males than females in F_1_ male backcross progeny of the resistant Ankara strain and a susceptible marker strain. The resistance was linked to autosomes II and V, and the strain was II^M^ (Kence and Kence 1992). These authors suggested that the linkage of M in the Ankara strain had shifted from III^M^ to II^M^ as a result of the malathion selection. It is therefore conceivable that an autosomal M factor could invade a natural population through linkage with an insecticide resistance allele, but selection for insecticide resistance cannot explain most of the autosomal M polymorphisms.

The geographical variation in the distribution of A^M^ and Y^M^ suggests that selection may be acting on fitness differences associated with different M genotypes in different environments. Fitness can be used to describe a variety of characteristics including, but not limited to, fecundity, emergence time, mating success, size, longevity, or susceptibility to disease. Deviations from random mating can be attributed to a difference in female receptivity, preferential mating within strains, or in male competition. There are important aspects of house fly biology that pertain to fitness of Y^M^, X^M^, and A^M^ males. House flies can survive the winter in cold climates as small populations living indoors, especially at livestock facilities (Keiding 1986). Black and Krafsur (1986) looked at seasonal house fly reproduction at one dairy and three swine farrowing sheds. They found slowed reproduction at the dairy in winter and early spring due to chronically low temperatures. House flies cannot survive freezing temperatures and do not diapause (Black and Krafsur 1986; Keiding 1986). House fly overwintering sites must offer microhabitats that remain greater than −5° with sufficient time greater than 10° (Rosales *et al.* 1994). Adults will mate within the first day after eclosion if adequate food is available (Milani 1975). The average mating speed for single pair crosses was found to be about 30 min and copulation lasts more than 1 hr (Bryant 1980). Females will only mate once unless additional sperm are necessary for further egg production (Keiding 1986). A recopulation frequency of 3.7% was determined (Baldwin and Bryant 1981). Genotype by environment fitness effects associated with any of these aspects of house fly biology could be responsible for the invasion of A^M^ and/or the maintenance of spatial gradients.

The relative fitness of Y^M^ *vs.* III^M^ males has been compared with the use of isogenic strains that carried the III^M^ or Y^M^ chromosome (Hamm *et al.* 2009). Three different comparisons were made. First, cages were started with 50% Y^M^ and 50% III^M^ males, and the frequencies of Y^M^ and III^M^ males were evaluated across generations. Second, mating competition studies were performed. Third, the relative emergence rates of III^M^ *vs.* Y^M^ pupae were examined at four temperatures. All three studies found that III^M^ males had a greater fitness than Y^M^ males. In the cage competition studies, >90% of the males were III^M^ after seven generations. III^M^ males were more likely to mate than Y^M^ males, and a greater percent of III^M^ males emerged after being held as pupae at 4, 16, or 28° for 3 d (Hamm *et al.* 2009).

In contrast to the aforementioned experiments, a comparison of the frequency of A^M^ and Y^M^ males in houseflies after 4 yr in the laboratory found a selective disadvantage for III^M^ males (Hamm and Scott 2008). In 2002, 77.7% of the male house flies were III/III;XY^M^, 20% were III^M^/III;XX, and 2.3% were III^M^/III;XY^M^ (karyotypes were inferred, not determined). After 4 yr in the laboratory, III^M^/III males disappeared and all of the males were either XY^M^ (82.6%) or X^M^Y^M^ (17.4%). There are at least four possible explanations why there was strong selection against III^M^ males in this laboratory experiment (Hamm and Scott 2008), but selection in favor of the III^M^ chromosome in the studies using the isogenic strains (Hamm *et al.* 2009): 1) the field collected strain that was left in the laboratory for 4 yr (Hamm and Scott 2008) contained *Md-tra^D^*; 2) there were four male genotypes in the 2008 study, but only two in the 2009 study (thus, the competition was not exactly the same); 3) the two papers used strains with different genetic backgrounds, which could influence the relative fitness; and 4) the 2008 study, as a whole, was not replicated.

The availability of the house fly genome sequence (Scott *et al.* 2014a) will open up new avenues of experimentation to further pursue fitness differences between Y^M^ and A^M^ males. For example, there is evidence for gene expression differences between Y^M^ and III^M^ males, which could be responsible for phenotypic differences that may be under selection (R. P. Meisel, J. G. Scott, and A. G. Clark, unpublished data).

### Why are there A^M^ and Y^M^ populations?

Ever since the discovery of differences between populations in the frequencies of A^M^ and Y^M^ males, researchers have struggled to understand the forces responsible for the patterns observed. Understanding the factors responsible for the invasion of new male- and female-determining loci in house fly and the maintenance of polygenic sex determination could reveal generalizable insights into the factors responsible for the evolution of sex determination.

The north-south clines (A^M^ in the south and Y^M^ in the north) observed in the Northern hemisphere (Franco *et al.* 1982; Tomita and Wada 1989b; Hamm *et al.* 2005) and the southern hemisphere (Y^M^ in the south and A^M^ in the north) (Feldmeyer *et al.* 2008) are best explained by seasonality in temperature variation, whereas variation in *Md-tra^D^* is best explained by variation in humidity and yearly mean temperature (Feldmeyer *et al.* 2008). This suggests that autosomal M factors may be linked to allelic variation with ecologically adaptive fitness effects. Other types of clines also have been observed (*e.g.*, radial), which suggests additional environmental variables may be associated with the distribution of A^M^ and Y^M^. Although it also was hypothesized that increases in A^M^ could be correlated with insecticide resistance (Hiroyoshi 1980; Franco *et al.* 1982; Kence and Kence 1992), this does not appear to be the case for reasons discussed above.

The theoretical model of Bull and Charnov (1977) suggests two stages for the transition between standard populations (XY^M^ males and XX females) and populations fixed for an autosomal male determining locus (A^M^). In the first stage, an invading autosomal male determiner either confers a fitness benefit or is genetically linked to a beneficial allele, and it increases in frequency. In the second stage, an epistatic female determining factor (*e.g.*, *Md-tra^D^*) invades and allows for the fixation of the autosomal male determiner.

Bull and Charnov (1977) only modeled the invasion of new sex determining loci via natural selection, and they did not consider the role of sex ratio selection in the invasion of the female determining locus. However, subsequent work demonstrated that sex ratio selection could not cause a complete transition between sex determination systems in house fly, but it can affect the frequency of sex determining loci in populations (Kozielska *et al.* 2006). In addition, the Bull and Charnov (1977) model predicts equilibria in which polygenic sex determination is maintained (*i.e.*, the A^M^ locus does not fix). The altitudinal, latitudinal, and radial variation in A^M^ frequencies could be interpreted as either populations at a polygenic equilibrium or transient states on the way to fixation of A^M^. If these populations are on the way to fixation of A^M^, the relative stability of populations over generations suggests that this process is moving slowly.

## Unanswered questions and future directions

The complexity of sex determination in the house fly has left several unanswered questions. Many areas have not been considered or tested. Are autosomal M factors moving through populations because of a selective advantage? If a selective advantage is present, what phenotypes are under selection? Is selection acting directly on the phenotypic effects of different M and *Md-tra* alleles/loci, or does selection act on allelic variants genetically linked to the M or *Md-tra* loci? It is important to determine what the selection pressures are and how they vary in different environments, leading to populations that have varied frequencies of *Md-tr*a^D^, M factors and linkage of M. These results would allow us to test models for the evolution of sex determination, providing novel insights into the factors responsible for the evolution of sex determination pathways.

It is surprising that the linkage of M and frequency of females with *Md-tra^D^* has not been investigated in Central or South America. This is a gap in our knowledge that would be useful to fill because it would provide an additional independent test of geographic clines in the frequency of A^M^. In addition, there are relatively few studies that have determined the frequency of males with multiple M factors and/or the frequency of females with *Md-tra^D^*. More studies of this type will help understand how these genes co-evolve.

What is M? Identification of the M factor would be a tremendous advance for understanding house fly sex determination and the nature of sex determination pathways in general. Is the M factor a mobile element that can transpose between chromosomes or is each instance of M on a different chromosome a unique gene that has gained the ability to negatively regulate *Md-tra*? Is the same gene used in other dipteran sex determination pathways as a male determining locus? Knowing the identity of M would allow us to test whether the variability in the frequency of M on different chromosomes is a result of fitness effects of different alleles of M on each chromosome or selection on allelic variation in genes linked to the autosomal M loci.

Although several studies have found populations in which M is not linked to an autosome, clarification as to whether such populations are Y^M^ or X^M^ would be helpful. If we knew the sequence of M, we also could potentially identify chromosome-specific allelic variants of M. That would allow us to diagnose the location of M through genotyping by sequencing (potentially being able to diagnose the locations of multiple M factors in an individual as well). We could then sample old specimens (*e.g.*, from insect collections) to shed light on the relative frequency of M on different chromosomes in populations from the 1800s and 1900s. Efforts to karyotype house flies are laborious and substantial time must be spent learning how to correctly assess the patterns of chromosomes in the squashes. Having visible labels, stains, or molecular markers for specific chromosomes, especially X and Y, would greatly facilitate obtaining the proper karyotype (and would move this area of investigation forward at a more rapid pace).

The genome sequence of the house fly will allow for investigations that test for early differentiation of nascent sex chromosomes. Studying such “neo-X” and “neo-Y” chromosomes has been a fruitful area of research in *Drosophila* genetics to characterize the evolutionary forces that act upon X and Y chromosomes (Sturgill *et al.* 2007; Meisel *et al.* 2009; Zhou and Bachtrog 2012). There are many theoretical predictions about how mutation, selection, recombination, and genetic drift drive the differentiation of sex chromosomes (Vicoso and Charlesworth 2006), and the house fly is poised to be a unique model for investigating the early stages of this important evolutionary process.

The house fly is a serious threat to human and animal health. Adult house flies are vectors of more than 100 human and animal intestinal diseases (Scott and Lettig 1962; Greenberg 1965; Keiding 1986). They are capable of transmitting parasites that cause typhoid fever, cholera, bacillary dysentery, infantile diarrhea, tuberculosis, plague, leprosy, yaws, salmonellosis, anthrax, and other diseases (West 1951). Flies also transmit eye diseases such as trachoma and epidemic conjunctivitis (Keiding 1986). Therefore, control of house flies is an area of great significance, but most approaches rely on the use of insecticides which present environmental and health concerns. Release of sterile males has been a great success for some Diptera, such as screw worm (*Cochliomyia hominivorax*) (Knipling 1960). An understanding of the factors underlying the relative frequency of Y^M^ and A^M^, as well as the identification of M may offer new insights into fly reproduction that could lead to new control methods, such as the release of homozygous sterile M males into closed systems, such as poultry facilities. This would lead the following generation to produce all males, providing control of the population. Additional strategies will follow as a deeper understanding of this biological system is attained.

## Supplementary Material

Supporting Information

## References

[bib1] Baker R. H. Sakai R. K., 1976 Male determining factor on chromosome 3 in the mosquito, *Culex tritaeniorhynchus*. J. Hered. 67: 289–294. 1010930 10.1093/oxfordjournals.jhered.a108733

[bib2] Baldwin F. T. Bryant E. H., 1981 Effect of size upon mating performance within geographic strains of the housefly, *Musca domestica* L. Evolution 35: 1134–1141. 10.1111/j.1558-5646.1981.tb04984.x 28563386

[bib3] Black W. C. Krafsur E. S., 1986 Seasonal breeding structure in house fly, *Musca domestica* L., populations. Heredity 56: 289–298.

[bib4] Boggs R. T. Gregor P. Idriss S. Belote J. M. McKeown M., 1987 Regulation of sexual differentiation in *D. melanogaster* via alternative splicing of RNA from the *transformer* gene. Cell 50: 739–747. 2441872 10.1016/0092-8674(87)90332-1

[bib5] Bopp D., 2010 About females and males: continuity and discontinuity in flies. J. Genet. 89: 315–323. 20876998 10.1007/s12041-010-0043-9

[bib6] Bopp D. Saccone G. Beye M., 2014 Sex determination in insects: variations on a common theme. Sex Dev. 8: 20–28. 24335049 10.1159/000356458

[bib7] Boyes J. W. Corey M. J. Paterson H. E., 1964 Somatic chromosomes of higher diptera, ix. karyotypes of some muscid species. Can. J. Zool. 42: 1025–1036.

[bib8] Boyes J. W. van Brink J. M., 1965 Chromosomes of calyptrate diptera. Can. J. Genet. Cytol. 7: 537–550.

[bib9] Bridges C. B., 1921 Triploid intersexes in *Drosophila melanogaster*. Science 54: 252–254. 17769897 10.1126/science.54.1394.252

[bib10] Bryant E. H., 1980 Geographic variation in components of mating success of the housefly, *Musca domestica L*., in the United States. Am. Nat. 116: 655–669.

[bib11] Bull J. J., 1983 Evolution of Sex Determining Mechanisms. Benjamin/Cummings, Menlo Park, CA.

[bib12] Bull J. J. Charnov E. L., 1977 Changes in the heterogametic mechanism of sex determination. Heredity 39: 1–14. 268319 10.1038/hdy.1977.38

[bib13] Bulmer M. G. Bull J. J., 1982 Models of polygenic sex determination and sex ratio control. Evolution 36: 13–26. 10.1111/j.1558-5646.1982.tb05005.x 28581110

[bib14] Burghardt G. Hediger M. Siegenthaler C. Moser M. Dubendorfer A., 2005 The *transformer2* gene in *Musca domestica* is required for selecting and maintaining the female pathway of development. Dev. Genes Evol. 215: 165–176. 15662529 10.1007/s00427-004-0464-7

[bib15] Cakir S., 1999 Two new sex determining factors (M^V^, F^D^) in housefly, (*Musca domestica*) populations in Turkey. Turkish J. Zool. 23: 73–77.

[bib16] Cakir S. Kence A., 1996 The distribution of males having XY and XX chromosomes in housefly populations (Diptera: Muscidae) of Turkey. Genetica 98: 205–210.

[bib17] Cakir S. Kence A., 2000 Polymorphism of M factors in populations of the housefly, *Musca domestica* L., in Turkey. Genet. Res. 76: 19–25. 11006631 10.1017/s0016672300004596

[bib18] Casper A. L. Van Doren M., 2009 The establishment of sexual identity in the *Drosophila* germline. Development 136: 3821–3830. 19855024 10.1242/dev.042374 PMC2766343

[bib19] Charlesworth D. Charlesworth B., 1980 Sex differences in fitness and selection for centric fusions between sex-chromosomes and autosomes. Genet. Res. 35: 205–214. 6930353 10.1017/s0016672300014051

[bib20] Cline T. W., 1984 Autoregulatory functioning of a *Drosophila* gene product that establishes and maintains the sexually determined state. Genetics 107: 231–277. 6735170 10.1093/genetics/107.2.231 PMC1202321

[bib21] Cline T. W., 1988 Evidence That *sisterless-a* and *sisterless-b* are two of several discrete “numerator elements” of the X/A sex determination signal in *Drosophila* that switch *Sxl* between two alternative stable expression states. Genetics 119: 829–862. 3137120 10.1093/genetics/119.4.829 PMC1203469

[bib22] Concha C. Scott M. J., 2009 Sexual development in *Lucilia cuprina* (Diptera, Calliphoridae) is controlled by the *transformer* gene. Genetics 182: 785–798. 19433631 10.1534/genetics.109.100982 PMC2710159

[bib23] DeFalco T. Camara N. Le Bras S. Van Doren M., 2008 Nonautonomous sex determination controls sexually dimorphic development of the *Drosophila* gonad. Dev. Cell 14: 275–286. 18267095 10.1016/j.devcel.2007.12.005 PMC2292836

[bib24] Demir E. Dickson B. J., 2005 *fruitless* splicing specifies male courtship behavior in *Drosophila*. Cell 121: 785–794. 15935764 10.1016/j.cell.2005.04.027

[bib25] Denholm I. Franco M. G. Rubini P. G. Vecchi M., 1983 Identification of a male determinant on the X chromosome of housefly (*Musca domestica* L.) populations in South-East England. Genet. Res. 42: 311–322.

[bib26] Denholm I. Franco M. G. Rubini P. G. Vecchi M., 1985 Geographical variation in house-fly (*Musca domestica* L.) sex determinants within the British Isles. Genet. Res. 47: 19–27.

[bib27] Denholm I. Rubini P. G. Rovati C. Vecchi M., 1990 Genetic basis of sex determination in two South African strains of house fly. S. Afr. J. Sci. 86: 41–43.

[bib28] Dübendorfer A., 2001 Genetic control of sex determination in the housefly, pp. 190–197 in Encyclopedia of Genetics, edited by Reeve E. C. R., Fritzroy Dearborn, London.

[bib29] Dübendorfer A. Hediger M., 1998 The female-determining gene *F* of the housefly, *Musca domestica*, acts maternally to regulate its own zygotic activity. Genetics 150: 221–226. 9725841 10.1093/genetics/150.1.221 PMC1460308

[bib30] Dübendorfer A. Hediger M. Burghardt G. Bopp D., 2002 *Musca domestica*, a window on the evolution of sex-determining mechanisms in insects. Int. J. Dev. Biol. 46: 75–79. 11902690

[bib31] Duffy J. B. Gergen J. P., 1991 The *Drosophila* segmentation gene runt acts as a position-specific numerator element necessary for the uniform expression of the sex-determining gene *Sex-lethal*. Genes Dev. 5: 2176–2187. 1748277 10.1101/gad.5.12a.2176

[bib32] Erickson J. W. Quintero J. J., 2007 Indirect effects of ploidy suggest X chromosome dose, not the X:A ratio, signals sex in *Drosophila*. PLoS Biol. 5: e332. 18162044 10.1371/journal.pbio.0050332 PMC2222971

[bib33] Eshel I., 1975 Selection on sex-ratio and the evolution of sex-determination. Heredity 34: 351–361. 1056322 10.1038/hdy.1975.44

[bib142] Este S. V. Rovati C., 1982 Inheritance of the arrhenogenic factor *Ag* of *Musca domestica* L. Boll. Zool. 49: 269–278.

[bib34] Feldmeyer B. Pen I. Beukeboom L. W., 2010 A microsatellite marker linkage map of the housefly, *Musca domestica*: evidence for male recombination. Insect Mol. Biol. 19: 575–581. 20491981 10.1111/j.1365-2583.2010.01016.x

[bib35] Feldmeyer B. Kozielska M. Kuijper B. Weissing F. Beukeboom L., 2008 Climatic variation and the geographical distribution of sex-determining mechanisms in the housefly. Evol. Ecol. Res. 10: 797–809.

[bib36] Fisher R. A., 1930 The Genetical Theory of Natural Selection. Clarendon Press, Oxford.

[bib37] Franco M. G. Rubini P. G. Vecchi M., 1982 Sex-determinants and their distribution in various populations of *Musca domestica* L. of Western Europe. Genet. Res. 40: 279–293. 7160618 10.1017/s0016672300019157

[bib38] Gethmann R. C., 1988 Crossing over in males of higher diptera (Brachycera). J. Hered. 79: 344–350. 10.1093/oxfordjournals.jhered.a110526 31581763

[bib39] Geuverink E. Beukeboom L. W., 2014 Phylogenetic distribution and evolutionary dynamics of the sex determination genes doublesex and transformer in insects. Sex Dev. 8: 38–49. 24401160 10.1159/000357056

[bib40] Goodenough J. McGuire B. Wallace R., 1993 Sexual reproduction and sexual selection, pp. 447–489 in Perspectives on Animal Behavior, edited by Cheney S., John Wiley & Sons, Inc., New York.

[bib41] Graham P. Penn J. K. M. Schedl P., 2003 Masters change, slaves remain. BioEssays 25: 1–4. 12508274 10.1002/bies.10207

[bib42] Greenberg B., 1965 Flies and disease. Sci. Am. 213: 92–99. 14298724 10.1038/scientificamerican0765-92

[bib43] Haag E. S. Doty A. V., 2005 Sex determination across evolution: connecting the dots. PLoS Biol. 3: e21. 15660158 10.1371/journal.pbio.0030021 PMC544544

[bib44] Hamilton W. D., 1967 Extraordinary sex ratios. Science 156: 477–488. 6021675 10.1126/science.156.3774.477

[bib45] Hamm, R. L., 2008 Exploring the population genetics of the house fly sex determining genes, M and F. Ph.D. Thesis, Cornell University, Ithaca, NY.

[bib46] Hamm R. L. Scott J. G., 2008 Changes in the frequency of Y^M^ *vs.* III^M^ in the house fly, *Musca domestica* L., under field and laboratory conditions. Genet. Res. 90: 1–6. 10.1017/S0016672308009853 19123967

[bib47] Hamm R. L. Scott J. G., 2009 A high frequency of male determining factors in male *Musca domestica* L. (Diptera: Muscidae) from Ipswich, Australia. J. Med. Entomol. 46: 169–172. 19198531 10.1603/033.046.0121

[bib48] Hamm R. Shono T. Scott J. G., 2005 A cline in frequency of autosomal males is not associated with insecticide resistance in house fly (Diptera: Muscidae). J. Econ. Entomol. 98: 171–176. 15765679 10.1093/jee/98.1.171

[bib49] Hamm R. L. Gao J.-R. Lin G. G.-H. Scott J. G., 2009 Selective advantage for III^M^ males over Y^M^ males in cage competition, mating competition and pupal emergence in *Musca domestica* L. (Diptera: Muscidae). Environ. Entomol. 38: 499–504. 19389301 10.1603/022.038.0225

[bib50] Hediger M. Minet A. D. Niessen M. Schmidt R. Hilfiker-Kleiner D., 1998 The male-determining activity on the *Y* chromosome of the housefly (*Musca domestica* L.) consists of separable elements. Genetics 150: 651–661. 9755197 10.1093/genetics/150.2.651 PMC1460372

[bib51] Hediger M. Burghardt G. Siegenthaler C. Buser N. Hilfiker-Kleiner D., 2004 Sex determination in *Drosophila melanogaster* and *Musca domestica* converges at the level of the terminal regulator *doublesex*. Dev. Genes Evol. 214: 29–42. 14673649 10.1007/s00427-003-0372-2

[bib52] Hediger M. Henggeler C. Meier N. Perez R. Saccone G., 2010 Molecular characterization of the key switch *F* provides a basis for understanding the rapid divergence of the sex-determining pathway in the housefly. Genetics 184: 155–170. 19841093 10.1534/genetics.109.109249 PMC2815913

[bib53] Hilfiker-Kleiner D. Dubendorfer A. Hilfiker A. Nothiger R., 1994 Genetic control of sex determination in the germ line and soma of the housefly, *Musca domestica*. Development 120: 2531–2538. 7956829 10.1242/dev.120.9.2531

[bib54] Hiroyoshi T., 1964 Sex-limited inheritance and abnormal sex ratio in strains of the housefly. Genetics 50: 373–385. 14207704 10.1093/genetics/50.3.373 PMC1210658

[bib55] Hiroyoshi, T., 1980 Formal genetics of the housefly in relation to insecticide resistance, pp. 397 in *Proceedings of the 16th International Congress of Entomology*, Elsevier Biomedical, Kyoto, Japan.

[bib56] Hiroyoshi T. Fukumori Y. Inoue H., 1982 Male crossing-over and location of the male determining factor on the third chromosome in a III^M^-type strain of the housefly. Jpn. J. Genet. 57: 231–239.

[bib57] Hoehn K.B. Noor M.A.F., 2015 How big is your Y? A genome-sequence based estimate of the size of the male-specific region in *Megaselia scalaris*. G3 (Bethesda) 5: 45–48. 25380730 10.1534/g3.114.015057 PMC4291468

[bib59] Horsfall W. R. Anderson J. F., 1963 Thermally induced genital appendages on mosquitoes. Science 141: 1183–1184. 14043361 10.1126/science.141.3586.1183

[bib60] Hoshijima K. Inoue K. Higuchi I. Sakamoto H. Shimura Y., 1991 Control of doublesex alternative splicing by transformer and transformer-2 in *Drosophila*. Science 252: 833–836. 1902987 10.1126/science.1902987

[bib61] Inoue H. Hiroyoshi T., 1982 A male-determining factor on autosome 1 and occurrence of male-recombination in the housefly, *Musca domestica* L. Jpn. J. Genet. 57: 221–229.

[bib62] Inoue H. Hiroyoshi T., 1986 A maternal-effect sex-transformation mutant of the housefly, *Musca domestica* L. Genetics 112: 469–482. 17246316 10.1093/genetics/112.3.469 PMC1202758

[bib63] Inoue H. Fukumori Y. Hiroyoshi T., 1983 Mapping of autosomal male-determining factors of the housefly, *Musca domestica* L., by means of sex reversal. Jpn. J. Genet. 58: 451–461.

[bib64] Ito H. Fujitani K. Usui K. Shimizu-Nishikawa K. Tanaka S., 1996 Sexual orientation in *Drosophila* is altered by the *satori* mutation in the sex-determination gene *fruitless* that encodes a zinc finger protein with a BTB domain. Proc. Natl. Acad. Sci. USA 93: 9687–9692. 8790392 10.1073/pnas.93.18.9687 PMC38490

[bib65] Kavi L. A. K. Kaufman P. E. Scott J. G., 2014 Genetics and mechanisms of imidacloprid resistance in house flies. Pestic. Biochem. Physiol. 109: 64–69. 24581385 10.1016/j.pestbp.2014.01.006

[bib66] Keiding J., 1986 The House Fly: Biology And Control. World Health Organization (WHO), Vector Biology and Control Division, Geneva.

[bib67] Kence M. Kence A., 1992 Genetic consequences of linkage between malathion resistance and an autosomal male-determining factor in house fly (Diptera: Muscidae). J. Econ. Entomol. 85: 1566–1570. 1401476 10.1093/jee/85.5.1566

[bib69] Kerr R. W., 1960 Sex-limited DDT-resistance in house-flies. Nature 185: 868.

[bib71] Knipling E. F., 1960 The eradication of the screw-worm fly. Sci. Am. 203: 54–61. 13757129 10.1038/scientificamerican1060-54

[bib72] Konno Y. Scott J. G., 1991 Biochemistry and genetics of abamectin resistance in the house fly. Pestic. Biochem. Physiol. 41: 21–28.

[bib73] Kozielska M. Pen I. Beukeboom L. W. Weissing F. J., 2006 Sex ratio selection and multi-factorial sex determination in the housefly: a dynamic model. J. Evol. Biol. 19: 879–888. 16674584 10.1111/j.1420-9101.2005.01040.x

[bib74] Kozielska M. Feldmeyer B. Pen I. Weissing F. J. Beukeboom L. W., 2008 Are autosomal sex-determining factors of the housefly (*Musca domestica*) spreading north? Genet. Res. 90: 157–165. 10.1017/S001667230700907X 18426619

[bib75] Lagos D. Koukidou M. Savakis C. Komitopoulou K., 2007 The *transformer* gene in *Bactrocera oleae*: the genetic switch that determines its sex fate. Insect Mol. Biol. 16: 221–230. 17298554 10.1111/j.1365-2583.2006.00717.x

[bib77] Lester D. S. Crozier R. H. Shipp E., 1979 Recombination in the housefly, *Musca domestica*. Experientia 35: 175–176.

[bib79] Mainx F., 1964 The genetics of *Megaselia scalaris* Loew (Phoridae): a new type of sex determination in diptera. Am. Nat. 98: 415–430.

[bib80] Marin I. Baker B. S., 1998 The evolutionary dynamics of sex determination. Science 281: 1990–1994. 9748152 10.1126/science.281.5385.1990

[bib81] McDonald I. C., 1971 A male-producing strain of the house fly. Science 172: 489. 5550506 10.1126/science.172.3982.489

[bib82] McDonald I. C. Overland D. E., 1974 House fly genetics: variability in a field population. Ann. Entomol. Soc. Am. 67: 359–364.

[bib83] McDonald I. C. Overland D. E. Leopold R. A. Degrugillier M. E. Morgan P. B., 1975 Genetics of house flies: variability studies with North Dakota, Texas, and Florida populations. J. Hered. 66: 137–140. 1176759 10.1093/oxfordjournals.jhered.a108595

[bib84] McDonald I. C. Evenson P. Nickel C. A. Johnson O. A., 1978 House fly genetics: isolation of a female determining factor on chromosome 4. Ann. Entomol. Soc. Am. 71: 692–694.

[bib85] McKeown M. Belote J. M. Baker B. S., 1987 A molecular analysis of *transformer*, a gene in *Drosophila melanogaster* that controls female sexual differentiation. Cell 48: 489–499. 3100051 10.1016/0092-8674(87)90199-1

[bib86] Meier N. Käppeli S. Niessen M. Billeter J. Goodwin S., 2013 Genetic control of courtship behavior in the housefly: evidence for a conserved bifurcation of the sex-determining pathway. PLoS One 8: e62476. 23630634 10.1371/journal.pone.0062476 PMC3632534

[bib87] Meise M. Hilfiker-Kleiner D. Dübendorfer A. Brunner C. Nothiger R., 1998 *Sex-lethal*, the master sex-determining gene in *Drosophila*, is not sex-specifically regulated in *Musca domestica*. Development 125: 1487–1494. 9502729 10.1242/dev.125.8.1487

[bib88] Meisel R. P. Han M. V. Hahn M. W., 2009 A complex suite of forces drives gene traffic from *Drosophila* X chromosomes. Genome Biol. Evol. 1: 176–188. 20333188 10.1093/gbe/evp018 PMC2817413

[bib89] Milani R., 1975 The house fly, *Musca domestica*, pp. 377–399 in Handbook of Genetics, edited by King R. C. Plenum Press, New York.

[bib90] Milani R. Rubini P. G. Franco M. G., 1967 Sex-determination in the housefly. Genetica Agaria 21: 385–411.

[bib91] Pane A. Salvemini M. Bovi P. D. Polito C. Saccone G., 2002 The transformer gene in *Ceratitis capitata* provides a genetic basis for selecting and remembering the sexual fate. Development 129: 3715–3725. 12117820 10.1242/dev.129.15.3715

[bib92] Pane A. De Simone A. Saccone G. Polito C., 2005 Evolutionary conservation of *Ceratitis capitata transformer* gene function. Genetics 171: 615–624. 15998727 10.1534/genetics.105.041004 PMC1456775

[bib93] Pomiankowski A. Nothiger R. Wilkins A., 2004 The evolution of the *Drosophila* sex-determination pathway. Genetics 166: 1761–1773. 15126396 10.1534/genetics.166.4.1761 PMC1470811

[bib94] Raymond C. S. Shamu C. E. Shen M. M. Seifert K. J. Hirsch B., 1998 Evidence for evolutionary conservation of sex-determining genes. Nature 391: 691–695. 9490411 10.1038/35618

[bib95] Rice W. R., 1986 On the instability of polygenic sex determination: the effect of sex-specific selection. Evolution 40: 633–639. 10.1111/j.1558-5646.1986.tb00514.x 28556317

[bib96] Rosales A. L. Krafsur E. S. Kim Y., 1994 Cryobiology of the face fly and house fly (Diptera: Muscidae). J. Med. Entomol. 31: 671–680. 7966169 10.1093/jmedent/31.5.671

[bib97] Rubini P. G. Vecchi M. Franco M. G., 1980 Mitotic recombination in *Musca domestica* L. and its influence on mosaicism, gynandromorphism and recombination in males. Genet. Res. 35: 121–130.

[bib98] Ruiz M. F. Milano A. Salvemini M. Eirín-López J. M. Perondini A. L. P., 2007 The gene *transformer* of *Anastrepha* fruit flies (Diptera, Tephritidae) and its evolution in insects. PLoS ONE 2: e1239. 18043746 10.1371/journal.pone.0001239 PMC2080774

[bib99] Ryner L. C. Goodwin S. F. Castrillon D. H. Anand A. Villella A., 1996 Control of male sexual behavior and sexual orientation in *Drosophila* by the *fruitless* gene. Cell 87: 1079–1089. 8978612 10.1016/s0092-8674(00)81802-4

[bib100] Saccone G. Pane A. Polito L. C., 2002 Sex determination in flies, fruitflies and butterflies. Genetica 116: 15–23. 12484523 10.1023/a:1020903523907

[bib101] Salz H. K., 2011 Sex determination in insects: a binary decision based on alternative splicing. Curr. Opin. Genet. Dev. 21: 395–400. 21474300 10.1016/j.gde.2011.03.001 PMC3134629

[bib102] Salz H. K. Erickson J. W., 2010 Sex determination in *Drosophila*: the view from the top. Fly (Austin) 4: 60–70. 20160499 10.4161/fly.4.1.11277 PMC2855772

[bib103] Schmidt R. Hediger M. Nothiger R. Dübendorfer A., 1997 a The mutation *masculinizer* (*man*) defines a sex-determining gene with maternal and zygotic functions in *Musca domestica* L. Genetics 145: 173–183. 9017399 10.1093/genetics/145.1.173 PMC1207776

[bib104] Schmidt R. Hediger M. Roth S. Nothiger R. Dübendorfer A., 1997 b The *Y*-chromosomal and autosomal male-determining *M* factors of *Musca domestica* are equivalent. Genetics 147: 271–280. 9286686 10.1093/genetics/147.1.271 PMC1208110

[bib105] Schütt C. Nothiger R., 2000 Structure, function and evolution of sex-determining systems in Dipteran insects. Development 127: 667–677. 10648226 10.1242/dev.127.4.667

[bib106] Scott H. G. Lettig K. S., 1962 Flies of Public Health Importance and their Control. U.S. Government Printing Office, Washington, DC.

[bib109] Scott J. G. Shono T. Georghiou G. P., 1984 Genetic analysis of permethrin resistance in the house fly, *Musca domestica* L. Experientia 40: 1416–1418.

[bib110] Scott J. G. Warren W. C. Beukeboom L. W. Bopp D. Clark A. G., 2014 a Genome of the house fly (*Musca domestica* L), a global vector of diseases with adaptations to a septic environment. Genome Biol. 15: 466. 25315136 10.1186/s13059-014-0466-3 PMC4195910

[bib111] Scott M. J. Pimsler M. L. Tarone A. M., 2014 b Sex determination mechanisms in the Calliphoridae (blow flies). Sex Dev. 8: 29–37. 24401179 10.1159/000357132

[bib112] Sefton L. Timmer J. R. Zhang Y. Beranger F. Cline T. W., 2000 An extracellular activator of the *Drosophila* JAK/STAT pathway is a sex-determination signal element. Nature 405: 970–973. 10879541 10.1038/35016119

[bib113] Shearman D. C. A., 2002 The evolution of sex determination systems in dipteran insects other than *Drosophila*. Genetica 116: 25–43. 12484524 10.1023/a:1020955507978

[bib115] Shono T., 1985 Pyrethroid resistance: importance of the *kdr*-type mechanism. J. Pestic. Sci. 10: 141–146.

[bib118] Shono T. Scott J. G., 2003 Spinosad resistance in the house fly, *Musca domestica*, is due to a recessive factor on autosome 1. Pestic. Biochem. Physiol. 75: 1–7.

[bib120] Siegenthaler C. Maroy P. Hediger M. Dubendorfer A. Bopp D., 2009 Hormones and sex-specific transcription factors jointly control yolk protein synthesis in *Musca domestica*. Int. J. Evol. Biol. 2009: 291236. 21350653 10.4061/2009/291236 PMC3042604

[bib121] Stevens N. M., 1908 A study of the germ cells of certain Diptera with reference to the heterochromosomes and phenomenon of synapsis. J. Exp. Zool. 5: 359–374.

[bib122] Sturgill D. Zhang Y. Parisi M. Oliver B., 2007 Demasculinization of X chromosomes in the *Drosophila* genus. Nature 450: 238–241. 17994090 10.1038/nature06330 PMC2386140

[bib123] Sullivan R. L., 1961 Linkage and sex limitation of several loci in the housefly. J. Hered. 52: 282–286. 13918332 10.1093/oxfordjournals.jhered.a107097

[bib124] Tomita T. Wada Y., 1989 a Migration and linkage disequilibrium in local populations of the housefly (*Musca domestica*) in Japan. Jpn. J. Genet. 64: 383–389.

[bib125] Tomita T. Wada Y., 1989 b Multifactorial sex determination in natural populations of the housefly (*Musca domestica*) in Japan. Jpn. J. Genet. 64: 373–382.

[bib126] Traut W., 1994 Sex determination in the fly *Megaselia scalaris*, a model system for primary steps of sex chromosome evolution. Genetics 136: 1097–1104. 8005417 10.1093/genetics/136.3.1097 PMC1205866

[bib127] Traut W. Willhoeft U., 1990 A jumping sex determining factor in the fly *Megaselia scalaris*. Chromosoma 99: 407–412.

[bib128] Tsukamoto M., 1983 Methods of genetic analysis of insecticide resistance, pp. 71–98 in Pest Resistance to Pesticides, edited by Georghiou G. P. Saito T. Plenum Press, New York.

[bib129] Tsukamoto M. Shono T. Horio M., 1980 Autosomal sex-determining system of the housefly: discovery of the first-chromosomal male factor in Kitakyushu, Japan. J. Univ. Occup. Environ. Health 2: 235–252.

[bib130] Valcárcel J. Singh R. Zamore P. D. Green M. R., 1993 The protein Sex-lethal antagonizes the splicing factor U2AF to regulate alternative splicing of *transformer* pre-mRNA. Nature 362: 171–175. 7680770 10.1038/362171a0

[bib131] van Doorn G. S. Kirkpatrick M., 2007 Turnover of sex chromosomes induced by sexual conflict. Nature 449: 909–912. 17943130 10.1038/nature06178

[bib132] van Doorn G. S. Kirkpatrick M., 2010 Transitions between male and female heterogamety caused by sex-antagonistic selection. Genetics 186: 629–645. 20628036 10.1534/genetics.110.118596 PMC2954476

[bib134] Vicoso B. Charlesworth B., 2006 Evolution on the X chromosome: unusual patterns and processes. Nat. Rev. Genet. 7: 645–653. 16847464 10.1038/nrg1914

[bib135] Vicoso B. Bachtrog D., 2013 Reversal of an ancient sex chromosome to an autosome in *Drosophila*. Nature 499: 332–337. 23792562 10.1038/nature12235 PMC4120283

[bib136] Wagoner D. E., 1969 a Linkage group-karyotype correlation in the house fly, *Musca domestica* L., confirmed by cytological analysis of X-ray Induced Y-autosomal translocations. Genetics 62: 115–121. 5371008 10.1093/genetics/62.1.115 PMC1212255

[bib137] Wagoner D. E., 1969 b Presence of male determining factors found on three autosomes in the house fly, *Musca domestica*. Nature 223: 187–188. 5791727 10.1038/223187a0

[bib138] West L. S., 1951 The Housefly. Comstock Publishing, Ithaca, NY.

[bib139] White M. J. D., 1973 Animal Cytology and Evolution. Cambridge University Press, Cambridge.

[bib140] Wilkins A. S., 1995 Moving up the hierarchy: a hypothesis on the evolution of a genetic sex determination pathway. BioEssays 17: 71–77. 7702596 10.1002/bies.950170113

[bib141] Zhou Q. Bachtrog D., 2012 Sex-specific adaptation drives early sex chromosome evolution in *Drosophila*. Science 337: 341–345. 22822149 10.1126/science.1225385 PMC4107656

