# Supplementary material for: The Evolving Puzzle of Autosomal Versus Y-linked Male Determination in Musca domestica
Source: G3 (Bethesda). 2014 Dec 31;5(3):371–84. doi: 10.1534/g3.114.014795 (PMC4349091; doi:10.1534/g3.114.014795)
Supplement: Supporting Information [file supp_5_3_371__index.html]

The Evolving Puzzle of Autosomal Versus Y-linked Male Determination in Musca domestica — Supporting Information 

# The Evolving Puzzle of Autosomal *Versus* Y-linked Male Determination in *Musca domestica*

## Supporting Information for Hamm, Meisel, and Scott, 2015

**Files in this Data Supplement:**

- Table S1 - Linkage of M in laboratory strains of house fly. (PDF, 128 KB)
