# Supplementary material for: The Evolving Puzzle of Autosomal Versus Y-linked Male Determination in Musca domestica
Source: G3 (Bethesda). 2014 Dec 31;5(3):371–84. doi: 10.1534/g3.114.014795 (PMC4349091; doi:10.1534/g3.114.014795)
Supplement: Supporting Information [file supp_g3.114.014795_014795SI.pdf]

G3: Genes | Genomes | Genetics

The Evolving Puzzle of Autosomal Versus Y-linked Male Determination in *Musca domestica*

Ronda L. Hamm*, Richard P. Meisel[+] and Jeffrey G. Scott[#]

*Dow AgroSciences, Indianapolis, Indiana 46268

[+]Department of Biology and Biochemistry, University of Houston, Houston, Texas 77204

[#]Department of Entomology, Comstock Hall, Cornell University, Ithaca, New York 14853

Corresponding author:
Jeffrey G. Scott
Department of Entomology
6134 Comstock Hall
Cornell University
Ithaca, NY 14853
Phone: 607-255-7340
Email: jgs5@cornell.edu

DOI: 10.1534/g3.114.014795

Table S1 Linkage of M in laboratory strains of house fly

Strain (location experiments done)	Origin	Linkage of M	Reference
791spin	Denmark	III	(Højland *et al.* 2014)
A14-WHO	Turkey	II	(Kence and Kence 1993)
aabo	*	Y	(Franco *et al.* 1982)
aaby	*	Y	(Denholm *et al.* 1983)
aabys	*	Y^	(Scott 1989)
Akita-f	Japan	III	(Shono and Tsukamoto 1983)
ALHF (Alabama)	U.S.A.	Y/X	(Liu and Yue 2001)
Ankara	Turkey	III	(Kence and Kence 1992)
ASPR	Japan	III	(Shono and Scott 1990)
Cooper	*	Y	(Denholm *et al.* 1983)
CS	U.S.A.	III†	(Hamm *et al.* 2005)
DDT resistant	Australia	II	(Kerr 1970)
Gainesville	U.S.A.	III	(McDonald 1971)
KS8S3	U.S.A.	III	(Kavi *et al.* 2014)
LPR	U.S.A.	Y^	(Scott and Georghiou 1986)
Mutant strainII	*	III	(Cakir 1999)
Mutant strainIII	*	III	(Cakir 1999)
Nagai	Japan	III	(Hiroyoshi *et al.* 1982)
NYSPINR (New York)	U.S.A.	III	(Shono and Scott 1990)
SRS (Japan)	*	V	(Hamm *et al.* 2005)
SRS (Denmark)	*	III	(Hamm 2008)
SRS (Italy)	*	Y	(Franco *et al.* 1982)
Standard	*	Y	(Cakir 1999)
Wakamatsu-m	Japan	V	(Shono 1983)
WHO/IN	*	Y	(Kence and Kence 1993)
YBOL	Japan	III	(Lee and Shono 1996)
YPER	Japan	III†	(Shono *et al.* 2002)

Only strains where a definitive linkage of M was found are included in this table. Strains in which M was not linked to an autosome, but for which no karyotype information are available were labeled Y/X.
* Exact location of collection not specified.
^XY Karyotype determined by Hardstone and Scott (unpublished).
†XX Karyotype determined by Hardstone and Scott (unpublished).

LITERATURE CITED

Cakir, S., 1999 Two new sex determining factors (M^V, F^D) in housefly, (*Musca domestica*) populations in Turkey. Turkish J. Zool. 23: 73-77.

Denholm, I., M.G. Franco, P.G. Rubini, and M. Vecchi, 1983 Identification of a male determinant on the X chromosome of housefly (*Musca domestica* L.) populations in South-East England. Genet. Res. Camb. 42: 311-322.

Franco, M.G., P.G. Rubini, and M. Vecchi, 1982 Sex-determinants and their distribution in various populations of *Musca domestica* L. of Western Europe. Genet. Res. Camb. 40: 279-293.
Hamm, R., T. Shono, and J.G. Scott, 2005 A cline in frequency of autosomal males is not associated with insecticide resistance in house fly (Diptera: Muscidae). J. Econ. Entomol. 98: 171-176.
Hamm, R.L., 2008 *Exploring the population genetics of the house fly sex determining genes, M and F* Ithaca, NY: Cornell.
Hiroyoshi, T., Y. Fukumori, and H. Inoue, 1982 Male crossing-over and location of the male determining factor on the third chromosome in a III^{M}-type strain of the housefly. Jpn. J. Genet. 57: 231-239.
Højland, D.H., J.G. Scott, K.-M.V. Jensen, and M. Kristensen, 2014 Autosomal male determination in a spinosad resistant house fly strain from Denmark. Pest Man. Sci. 70: 1114-1117.
Kavi, L.A.K., P.E. Kaufman, and J.G. Scott, 2014 Genetics and mechanisms of imidacloprid resistance in house flies. Pestic. Biochem Physiol. 109: 64-69.
Kence, M., and A. Kence, 1992 Genetic consequences of linkage between malathion resistance and an autosomal male-determining factor in house fly (Diptera: Muscidae). J. Econ. Entomol. 85: 1566-1570.
Kence, M., and A. Kence, 1993 Control of insecticide resistance in laboratory populations of house fly (Diptera: Muscidea) by introduction of susceptibility genes. J. Econ. Entomol. 86: 189-194.
Kerr, R.W., 1970 Inheritance of DDT resistence in a laboratory colony of the housefly, *Musca Domestica*. Aust. J. Biol. Sci. 23: 377-400.
Lee, S., and T. Shono, 1996 Linkage group analysis of pyraclofos resistance in the housefly, *Musca domestica* L. Appl. Entomol. Zool. 31: 127-134.
Liu, N., and X. Yue, 2001 Genetics of pyrethroid resistance in a strain (ALHF) of house flies (Diptera: Muscidae). Pestic. Biochem. Physiol. 70: 151-158.
McDonald, I.C., 1971 A male-producing strain of the house fly. Science 172: 489.
Scott, J.G., 1989 Cross-resistance to the biological insecticide abamectin in pyrethroid resistant house flies. Pestic. Biochem. Physiol. 34: 27-31.
Scott, J.G., and G.P. Georghiou, 1986 The biochemical genetics of permethrin resistance in the Learn-PyR strain of house fly. Biochem. Genet. 24: 25-37.
Shono, T., 1983 Linkage group analysis of carboxylesterase in a malathion resistant strain of the housefly, *Musca domestica* L. (Diptera: Muscidae). Appl. Ent. Zool. 18: 407-415.
Shono, T., S. Kasai, E. Kamiya, Y. Kono, and J.G. Scott, 2002 Genetics and mechanisms of permethrin resistance in the YPER strain of house fly. Pestic. Biochem. Physiol. 73: 27-36.
Shono, T., and J.G. Scott, 1990 Autosomal sex-associated pyrethroid resistance in a strain of house fly (Diptera: Muscidae) with a male-determining factor on chromosome three. J. Econ. Entomol. 83: 686-689.
Shono, T., and M. Tsukamoto, 1983 Linkage group analysis of fenitrothion resistance in the housefly, *Musca domestica* L. (Diptera: Muscidae). App. Ent. Zool. 18: 98-105.
